# Metal–salen molecular cages as efficient and recyclable heterogeneous catalysts for cycloaddition of CO_2_ with epoxides under ambient conditions†
†Electronic supplementary information (ESI) available: Synthesis and characterization details, computational methods and supplementary catalytic data. See DOI: 10.1039/c8sc05019h


**DOI:** 10.1039/c8sc05019h

**Published:** 2018-11-29

**Authors:** Chee Koon Ng, Ren Wei Toh, Ting Ting Lin, He-Kuan Luo, T. S. Andy Hor, Jie Wu

**Affiliations:** a Department of Chemistry , National University of Singapore , 3 Science Drive 3 , Singapore 117543 , Singapore . Email: andyhor@hku.hk ; Email: chmjie@nus.edu.sg; b Institute of Materials Research and Engineering , Agency for Science, Technology and Research , #08-03, 2 Fusionopolis Way, Innovis , Singapore 138634 , Singapore; c Department of Chemistry , The University of Hong Kong , Pokfulam , Hong Kong SAR , China

## Abstract

A salen based molecular cage, salen@cage, was synthesized and complexed with Co and Al to yield metal–salen molecular cages, Co(ii)@cage, Co(iii)@cage and Al(iii)@cage. These cages were demonstrated to be efficient heterogeneous catalysts for the cycloaddition of CO_2_ with styrene oxide, achieving full conversion at 25 °C and 1 atm CO_2_. Good to excellent yields of various cyclic carbonates were also achieved under mild conditions. Al(iii)@cage can be reused up to five times without any significant loss of its high catalytic activity. The capability to access a variety of heterogeneous organometallic catalysts with salen@cage offers new prospects for practical CO_2_ utilization and chemical manufacturing.

## Abstract 2

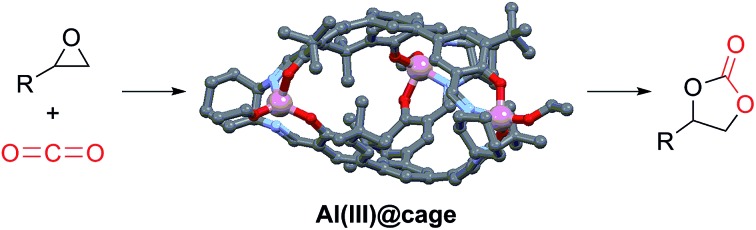
Metal-salen molecular cages are efficient and recyclable heterogeneous catalysts for cycloaddition of CO_2_, achieving full conversion at ambient conditions.

## Introduction

Molecular cages have been of much interest over the past decade as these materials have a wide range of applications from molecular recognition^1^ to chemical sensors.^2^ Moreover, their high surface area and porous structure allow them to find applications in gas separation and storage.^3^ The reactive sites found within these porous molecular cages are suitable for catalytic applications as they can be used to stabilize metal nanoparticles like Pd^4^ and Rh,^5^ increasing their catalytic activities towards organic transformations. Molecular cages can also trap both homogeneous catalysts and substrates *via* specific binding sites. This preorganization brings the encapsulated substrates and catalyst into a confined cavity which promotes the catalytic process.^6^

Salen ligands and their metal complexes have been well studied over the last two decades in homogeneous catalysis.^7^ These ligands are versatile as they can coordinate with many transition metals, main group metals, and even lanthanides. Their complexes have been employed as catalysts in a wide variety of organic transformations, *e.g.* epoxidation of alkenes, Diels–Alder reactions, oxidation, ring opening of epoxides, Michael addition and reduction of ketones.^8^ One of the most important application of these metal salen complexes is to catalyze the cycloaddition of CO_2_ with epoxides to form cyclic carbonates. The increasing anthropogenic emissions of CO_2_ have resulted in excessive global warming, and thus the efficient utilization of CO_2_ as a C_1_ source is an appealing subject of investigation.^9^ Co(iii) and Al(iii) salen complexes were effective catalysts for the cycloaddition of CO_2_ to epoxides and the reaction usually proceeds under mild conditions.^10^ The cyclic carbonate products obtained have a range of different applications, including being used as green solvents,^11^ electrolytes in lithium-ion batteries^12^ and precursors in organic synthesis as intermediates to important chemicals like glycols, polyurethanes, dialkyl carbonates, carbamates, purines and pyrimidines. Although homogeneous catalysts like Co(iii) and Al(iii) salen complexes^13^ were effective at coupling CO_2_ with epoxides,^14^ they suffer from poor catalyst separation and low recyclability. On the other hand, heterogeneous catalysts like metal oxides,^15^ supported metal complexes,^16^ metal organic frameworks (MOFs)^17^ and porous polymers^18^ have been investigated for the production of cyclic carbonates from CO_2_, but many of these catalysts require elevated temperatures and pressures or long reaction durations. Therefore, heterogenization of these metal salen complexes to obtain highly efficient catalysts is important not only for the CO_2_ cycloaddition, but also for shedding light on the diverse metal salen-mediated chemical transformations.^8^

We herein report that by utilizing dynamic imine condensation,^19^ molecular cages incorporated with the salen moiety (salen@cage) can be conveniently synthesized in moderate yields, which can undergo complexation with different metal precursors to give Co(ii)@cage, Co(iii)@cage and Al(iii)@cage. These cages show excellent catalytic reactivity for the cycloaddition of epoxide with CO_2_, giving full conversions of styrene oxide at room temperature and 1 atm CO_2_, probably because porous molecular cages serve to concentrate CO_2_ in the pores of the catalyst.

## Results and discussion

### Synthesis and characterization of salen@cage

**Scheme 1:**
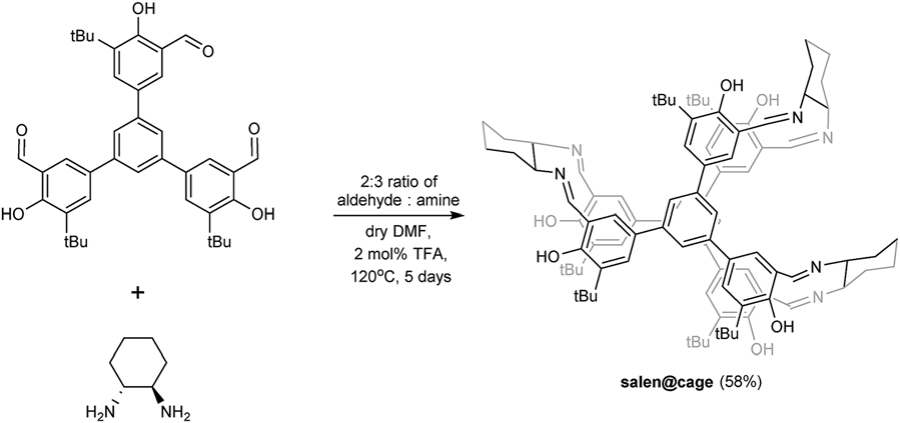
Synthesis of salen@cage.

The imine-based organic salen@cage was synthesized by the Schiff base reaction as shown in Scheme 1.^20^ In the ESI-MS spectrum, the salen@cage compound showed only one sharp signal at *m*/*z* 1447.83 with the expected isotopic pattern which corresponded to the cation of [2 + 3] salen@cage (Fig. S1 in the ESI†). MALDI-TOF MS in the *m*/*z* range of 750–8000 illustrated no further signals other than *m*/*z* 1448.0, which excluded the formation of smaller (*e.g.* [2 + 1] and [2 + 2]) or larger (*e.g.* [4 + 6] or up to [10 + 15]) condensation cages (Fig. S2 in the ESI†). The formation of salen@cage was further corroborated by high resolution mass spectroscopy, solid state ^13^C cross polarization magic angle spinning NMR and FT-IR (Fig. S3 and S4a in the ESI†). According to dynamic covalent chemistry, the reversible nature of the imine bonds, high reaction temperature and long reaction duration allowed for the most thermodynamically stable product to be selectively formed in equilibrium.19c,^21^ The [2 + 3] molecular prism^4^ was the most enthalpically favoured (least bond angle strain) and entropically favoured (least number of reactants).3*d* The use of other lower boiling point solvents (*e.g.* CHCl_3_ and THF) and shorter reaction durations resulted in the formation of smaller [2 + 2], [2 + 1] and [1 + 2] cages detected by ESI-MS.

### Synthesis and characterization of Co(ii)@cage, Co(iii)@cage and Al(iii)@cage

**Scheme 2:**
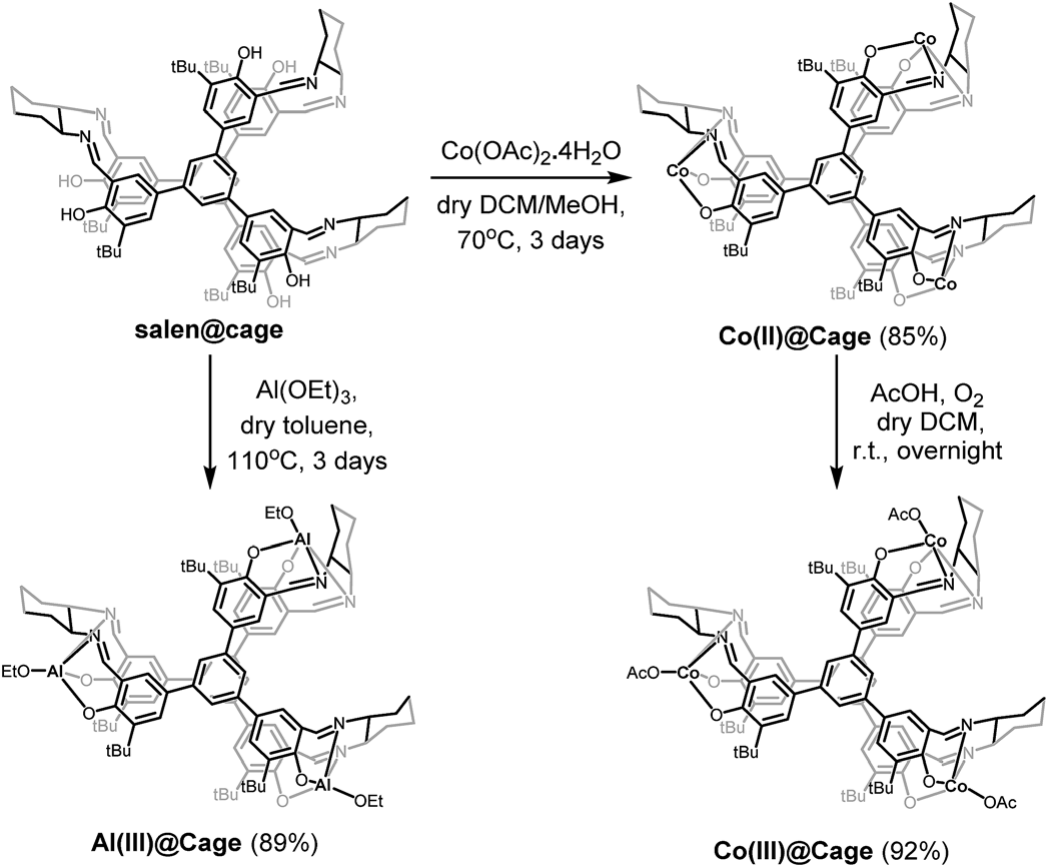
Synthesis of Co(ii)@cage, Co(iii)@cage and Al(iii)@cage.

Considering the high catalytic activity of Co and Al salen complexes in cycloaddition of CO_2_ with epoxides,13a,18a,^22^ Co and Al were subsequently coordinated onto salen@cage *via* reactions with Co(OAc)_2_ and Al(OEt)_3_, respectively (Scheme 2). Further purification of the compounds was done by Soxhlet extraction. Co(ii)@cage, Co(iii)@cage and Al(iii)@cage were all insoluble in common organic solvents, similar to salen@cage. Upon metalation, the characteristic C=N stretching of the imine bond shifts from 1630 cm^–1^ in salen@cage to 1607 cm^–1^ in Co(ii)@cage, 1609 cm^–1^ in Co(iii)@cage and 1627 cm^–1^ in Al(iii)@cage (Fig. S4b in the ESI†). The shifts to a lower frequency in FT-IR indicates that Co and Al have been coordinated onto the salen@cage.^23^ However, a small shoulder at around 1630 cm^–1^ can still be seen for Co(ii)@cage and Co(iii)@cage, which may suggest incomplete metalation for these two cage complexes which was confirmed by elemental analysis (Table S1 in the ESI†). In addition, the positive shifts of the N 1s peak in Co(ii)@cage, Co(iii)@cage and Al(iii)@cage as compared to salen@cage in XPS (Fig. S5, ESI†) corroborates the coordination of Co and Al onto the salen@cage.^24^ The Al 2p peak in the XPS spectrum (Fig. S6a, ESI†) indicates that aluminium is in the +3 oxidation state in Al(iii)@cage. The binding energies of the Co 2p peaks in the XPS spectrum (Fig. S6b, ESI†), together with the presence of observable satellite peaks,^25^ corroborate the +2 oxidation state of cobalt in Co(ii)@cage while the slightly higher binding energies of the Co 2p peaks (Fig. S6c, ESI†) and the absence of satellite peaks indicate that the cobalt in Co(iii)@cage is in the +3 oxidation state.^26^

We then studied the porosity and CO_2_ adsorption of these materials as these properties would affect the CO_2_ uptake and conversion. The porosity of these materials was studied by gas sorption experiments with N_2_ at 77 K and CO_2_ at 298 K (Fig. S9 and S10 in the ESI†). All the cages showed type I nitrogen gas adsorption isotherms according to the IUPAC classifications,^27^ indicating that these cage complexes consisted of both micropores and mesopores. DFT calculations of the molecular structure of Al(iii)@cage suggested that the micropores (<2 nm) likely originated from the intramolecular space within the cage compound (Fig. S11 in the ESI†), while the mesopores (2–50 nm) might have originated from the intermolecular packing between each cage molecule (Fig. S12 in the ESI†). All the synthesized cage complexes were considered to be moderately porous, with Al(iii)@cage displaying a higher BET surface area (771 m^2^ g^–1^) as compared to the other cage complexes (610–635 m^2^ g^–1^) (Table S2 in the ESI†). The CO_2_ adsorption capability of salen@cage (35.8 mg g^–1^) increased once it was coordinated to a metal (Co or Al). This was likely due to the Lewis acidic metal sites which polarized CO_2_ and led to greater adsorption.^28^ Due to the larger surface area, Al(iii)@cage displayed a larger CO_2_ adsorption capability (70.4 mg g^–1^) as compared to the rest of the cage complexes (35.8–49.2 mg g^–1^), which was comparable to that of the conjugated microporous polymers synthesized by Deng *et al.*18*a*

### Cycloaddition of CO_2_ with epoxides

We then investigated the cycloaddition of styrene oxide (SO) with CO_2_ to give styrene carbonate (SC) using our cage complexes at 25 °C and 1 atm CO_2_. Only 9% conversion was achieved after 24 hours in the absence of a catalyst (entry 1, Table 1). Using salen@cage as a catalyst did not improve the conversion (8%, entry 2). This proved that the metal centres in the metal cages were the active catalytic sites and the salen@cage served as the framework to support the metal salen complexes and to increase the surface area of the catalyst for the efficient diffusion of substrates (CO_2_ and SO). The heterogeneous Co(iii)@cage proved to be more active than Co(ii)@cage (100% *vs.* 55%, entries 4 and 3 respectively), because the more electrophilic Co(iii) could coordinate strongly with the epoxide and activated it for ring opening.13*a* Co(iii)@cage was also more efficient than Al(iii)@cage (100% *vs.* 75%, entries 4 and 7 respectively), although Al(iii)@cage could also achieve full conversion after 48 h (entry 8). DFT calculations by Deng and co-workers suggested that the Co–salen catalysts gave better catalytic activity as compared to the Al–salen catalysts due to the lower activation barrier of the Co–salen catalysts.^29^ When the catalyst loading of Co(iii)@cage was reduced to 0.17 mol%, the conversion of SO to SC decreased to 76% (entry 5). A longer reaction duration (48 instead of 24 h) at this reduced catalyst loading could achieve 98% conversion (entry 6). The homogeneous metal salts Co(OAc)_2_·4H_2_O and Al(OEt)_3_ exhibited much lower reactivity (59% and 24%, entries 9 and 10) as compared to the heterogeneous Co(iii)@cage and Al(iii)@cage. Previous reports on utilizing molecular cage complexes for CO_2_ cycloaddition are rare, although some molecular cages showed selective uptake of CO_2_ 20,30 and were able to trap CO_2_ as carbonate anions within the cage framework.^31^ In 2014, Martinez and Dufaud demonstrated that an azaphosphatrane–hemicryptophane cage complex was able to catalyze the CO_2_ cycloaddition to give styrene carbonate in 82% yield at 100 °C and 1 atm CO_2_.^32^

**Table 1:** Catalyst screening for cycloaddition of CO_2_ with styrene oxide to produce styrene carbonate at 25 °C^*a*^

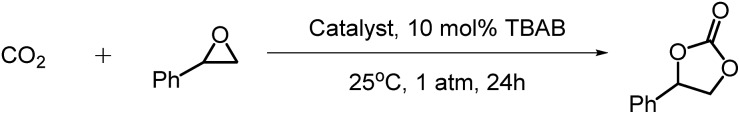			
Entry	Catalyst	Catalyst loading/mol%	Conversion^*b*^/%
1	NIL	NIL	9
2	salen@cage	0.33	8
3	Co(ii)@cage	0.33^*d*^	55
4	Co(iii)@cage	0.33^*d*^	>99
5	Co(iii)@cage	0.17^*e*^	76
6	Co(iii)@cage	0.17^*e*^	98^*c*^
7	Al(iii)@cage	0.33^*f*^	75
8	Al(iii)@cage	0.33^*f*^	>99^*c*^
9	Co(OAc)_2_·4H_2_O	1	59
10	Al(OEt)_3_	1	24

^*a*^Typical reaction conditions: 5.0 mmol styrene oxide, 0.5 mmol TBAB, and catalyst under 1 atm CO_2_ pressure at 25 °C for 24 h.

^*b*^Conversions calculated from the crude ^1^H NMR spectra.

^*c*^Reaction time = 48 h.

^*d*^Corresponding to approximately 1 mol% of Co.

^*e*^Corresponding to approximately 0.5 mol% of Co.

^*f*^Corresponding to approximately 1 mol% of Al.

We also investigated the enantioselectivity of the CO_2_ cycloaddition as the metal cage complexes were synthesized with chiral (*S*,*S*)-*trans*-1,2-diaminocyclohexane, which might impart a chiral environment to the heterogeneous catalysts. A low selectivity (13.6% ee) was obtained during the cycloaddition of CO_2_ with propylene oxide. However, the ee decreased to 2.3% when styrene oxide was used as the substrate (Fig. S15 and S16, ESI†).22b,^33^

**Fig. 1:**
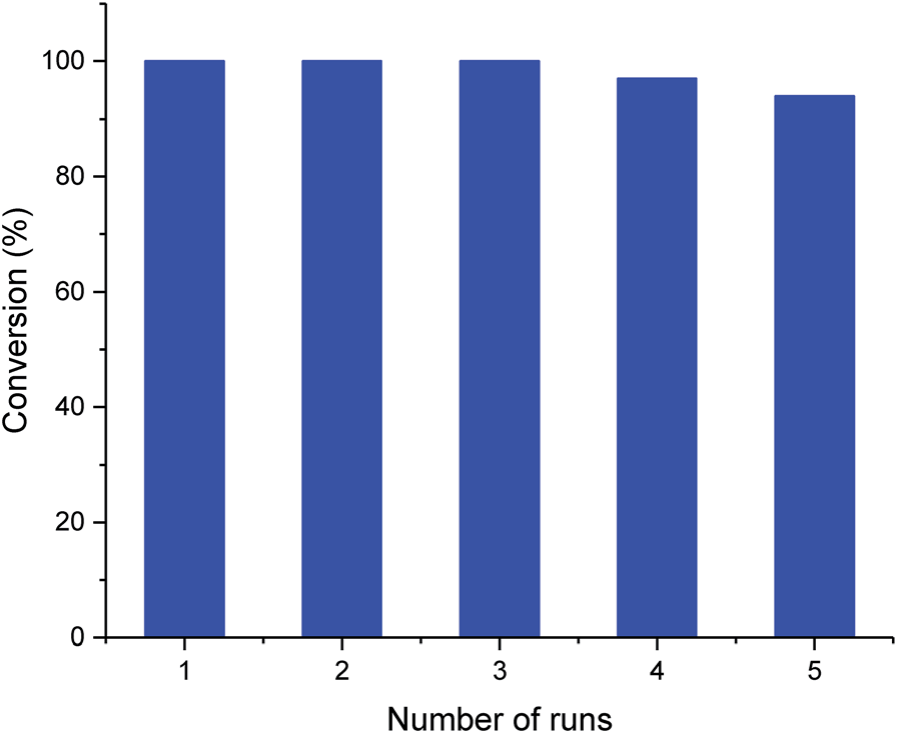
Recyclability of Al(iii)@cage. Typical reaction conditions: 5.0 mmol styrene oxide, 10 mol% TBAB and 0.33 mol% Al(iii)@cage under 1 atm CO_2_ pressure at 25 °C for 48 h.

**Fig. 2:**
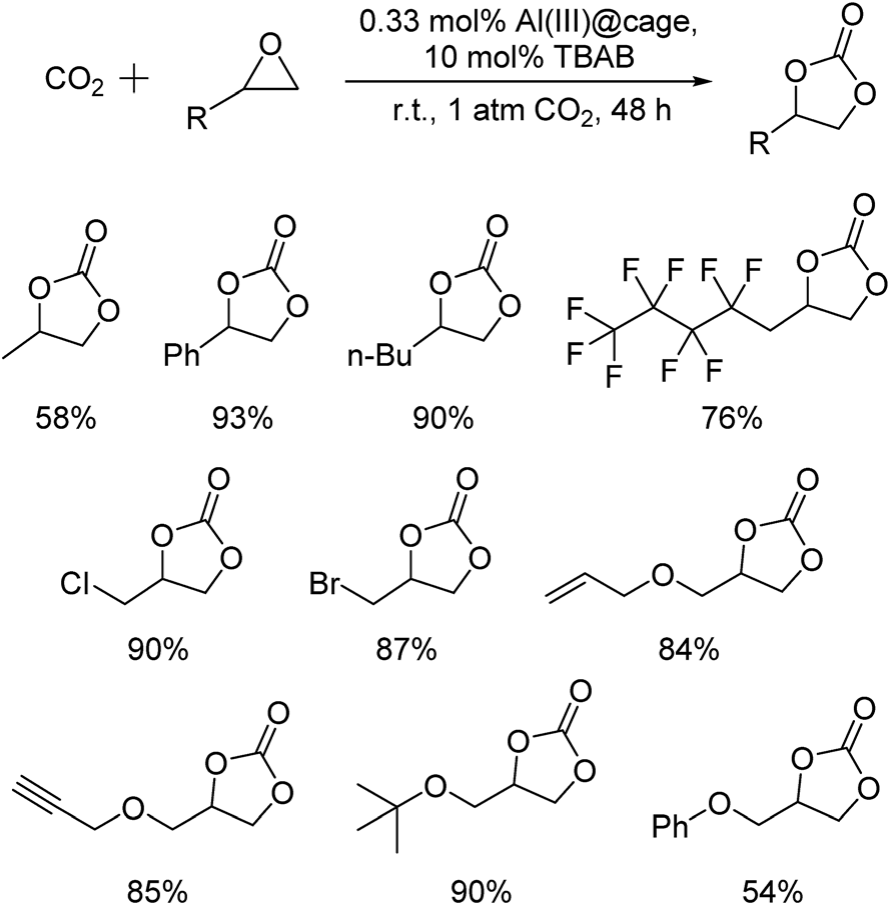
Isolated yields of cyclic carbonates formed by the cycloaddition of CO_2_ with epoxides catalyzed by Al(iii)@cage; reaction conditions: 1 mmol epoxide, 0.33 mol% Al(iii)@cage, 10 mol% TBAB, r.t., 1 atm CO_2_, and 48 h.

The recyclability of the metal cage complexes was subsequently evaluated. Al(iii)@cage retained most of its catalytic activity even after five runs, with the conversions dropping only slightly from 100% to 94% (Fig. 1). ICP-OES analysis of the reaction mixture after filtering shows only 52 ppm of Al, indicating negligible leaching of Al into the reaction mixture. However, Co(iii)@cage performed less convincingly in its recyclability testing, with conversions dropping from 100% to 67% (Fig. S17 in the ESI†). Finally, we investigated the epoxide scope with Al(iii)@cage under optimized conditions (Fig. 2). Good to excellent yields of the cyclic carbonates were achieved under mild conditions, illustrating that this catalytic system was effective with both alkyl and aryl epoxides, tolerating functionalities including halides, ethers, alkenes and alkynes. The modest yield for propylene carbonate was due to the high volatility of propylene oxide even at room temperature. In comparison with other high performing heterogeneous catalysts for this transformation, the catalytic activity of metal salen cages outperforms that of many supported metal complexes,16b–d,^34^, porous polymers^35^ and MOFs,^36^ but there have been recent reports of some MOFs^37^ and metalated porous organic polymers (POPs)18a,d–f,^38^ with similar or higher catalytic activities. The advantages of these cages include high stability, ease of synthesis, use of readily available materials, ability to adapt to different metals, and good recyclability with high productivity. Further optimisation can be focused on the control of porosity through molecular manipulation of the salen framework and its metal compatibility.

## Conclusions

We have successfully synthesized and characterized a salen-based [2 + 3] molecular cage, salen@cage. As a proof of concept for the heterogenization of metal salen complexes, Co and Al were coordinated onto the salen@cage to give Co(ii)@cage, Co(iii)@cage and Al(iii)@cage in excellent yields. Co(iii)@cage and Al(iii)@cage proved to be excellent heterogeneous catalysts for the cycloaddition of CO_2_ with styrene oxide, giving full conversions of styrene oxide to styrene carbonate at 25 °C and 1 atm CO_2_ with only 0.33 mol% catalyst loading. Al(iii)@cage could be reused up to five times without any significant decrease in its catalytic activity. The catalytic performances of molecular cage catalysts exceed that of many other heterogeneous CO_2_ cycloaddition catalysts under mild conditions which makes them suitable heterogeneous catalysts for CO_2_ conversion under ambient conditions. Moreover, our study opens the possibility of utilizing other metals with the salen@cage framework to yield a variety of efficient heterogeneous metal salen catalysts without the use of external heterogeneous supports like silica. These heterogeneous metal salen catalysts can potentially be applied in a variety of other transformations and also can be fitted for continuous-flow synthesis as a packed-bed catalyst. These studies are currently underway in our laboratory.

## Conflicts of interest

There are no conflicts to declare.

## Supplementary Material

Supplementary information
