# Supplementary material for: Metal–salen molecular cages as efficient and recyclable heterogeneous catalysts for cycloaddition of CO2 with epoxides under ambient conditions
Source: Chem Sci. 2018 Nov 29;10(5):1549–54. doi: 10.1039/c8sc05019h (PMC6357855; doi:10.1039/c8sc05019h)
Supplement: Supplementary file 1 [file SC-010-C8SC05019H-s001.pdf]

Electronic Supplementary Material (ESI) for Chemical Science.
This journal is © The Royal Society of Chemistry 2018

Supporting Information

Metal-salen molecular cages as efficient and recyclable heterogeneous catalysts for cycloaddition of CO_2 with epoxides at ambient conditions

Chee Koon Ng[a,b], Ren Wei Toh[a], a Ting Ting Lin[b], He-Kuan Luo[b], T.S. Andy Hor*[c], Jie Wu*[a]

[a] Department of Chemistry, National University of Singapore, 3 Science Drive 3, Singapore 117543, Singapore

[b] Institute of Materials Research and Engineering, Agency for Science, Technology and Research, #08-03, 2 Fusionopolis Way, Innovis, Singapore 138634, Singapore

[c] Department of Chemistry, The University of Hong Kong, Pokfulam, Hong Kong SAR, China.

Corresponding authors: Jie Wu (chmjie@nus.edu.sg)

T.S. Andy Hor (andyhor@hku.hk)

Contents

S1. General considerations S3

S2. Synthetic procedures and characterization details S5

S3. Catalytic cycloaddition of CO_2 with epoxides and catalyst recycling…………................S24

S4. Mechanism of cycloaddition of styrene oxide with CO_2..S28

S5. NMR of cyclic carbonates…………………………...S29

S6. Computational methods...S51

References: S62

S1. General considerations

General Procedure. All preparations and manipulations were performed using standard Schlenk techniques under a nitrogen atmosphere unless otherwise specified. Solvents were dried using activated 3Å molecular sieves or purchased from commercial suppliers. Chemicals were purchased from commercial suppliers and used as received without further purification.

Ligands (**A**) – (**D**) were synthesized according to literature procedures.[1] ^{1}H and ^{13}C solution NMR spectra were measured at 25°C using a JEOL Resonance 500MHz NMR spectrometer. Chemical shifts are expressed with a positive sign, in parts per million, relative to residual ^{1}H and ^{13}C solvent signals. Data are reported as follows: chemical shift, multiplicity (s = singlet, d = doublet, t = triplet, q = quartet, m = multiplet, br = broad), coupling constants (Hz) and integration. The ^{13}C CP-MAS NMR spectra were measured on a standard-bore Varian NMR System 400MHz spectrometer equipped with a 4 mm 1H/X CP-MAS probe. The Varian standard pulse program tancp was used with a rotational frequency of 12 kHz, a recycle delay of 3s with a radiofrequency (RF) field of 100 kHz. The spectrum was referenced to external Hydroxymethyl Butyrate (d=17.34ppm). Flash column chromatography was generally performed using Silica Gel. Elemental analyses were performed on a VarioMICRO Elementar system. Inductively coupled plasma optical emission spectroscopy was carried out on a Perkin Elmer Optima 5300DV instrument.

IR spectra were recorded using a Perkin-Elmer FTIR 2000 spectrometer using KBr pellets. ESI-MS measurements were performed on Finnigan LCQ mass spectrometer, isotope distribution patterns were used as a composition proof in addition to m/z signal. HR-MS was conducted on Bruker MicrOTOF-QII spectrometer. MALDI-TOF mass spectra (MS) were recorded on a Bruker Autoflex instrument using dithranol as matrix. Thermogravimetric

analysis (TGA) was performed on a TA Instruments Q500 thermogravimetric analyser by measuring the weight loss while heating at a rate of 10 °C min^{-1} from room temperature to 800 °C under nitrogen. Nitrogen sorption isotherms were measured at 77 K with an ASAP 2020 Automatic High Resolution Micropore Physisorption Analyser. Before measurement, the samples were degassed in vacuum at 100 °C for at least 10 h. The Brunauer−Emmett−Teller (BET) surface area was evaluated using N_2 adsorption data in the relative pressure (P/P_0) range of 0.01 - 0.14. Powder X-ray Diffraction (PXRD) patterns of samples were collected over the 2Θ range 5° to 90° on a Bruker AXS GADDS X-ray diffractometer with Cu-Ka radiation (λ = 1.54056 Å). The scanning electron micrographs (SEM) were taken with a JEOL FESEM JSM6700F. XPS spectra were measured on powder samples using VG Thermo Escalab 220i -XL X-ray photoelectron spectroscopy system and XPS data was analyzed using Thermo Avantage v4.12. The ee of propylene carbonate was determined via gas chromatography (Shimadzu GC 2010 plus) using Astec Chiraldex BTA with the column temperature set at 160°C (isothermal) while the detector and injector temperatures were set at 180°C. The ee of styrene carbonate was determined by HPLC (Shimadzu LC-20AD) using a Chiralcel OD-H column (eluent 80:20 Hexane:iPrOH).

S2. Synthetic procedures and characterization details

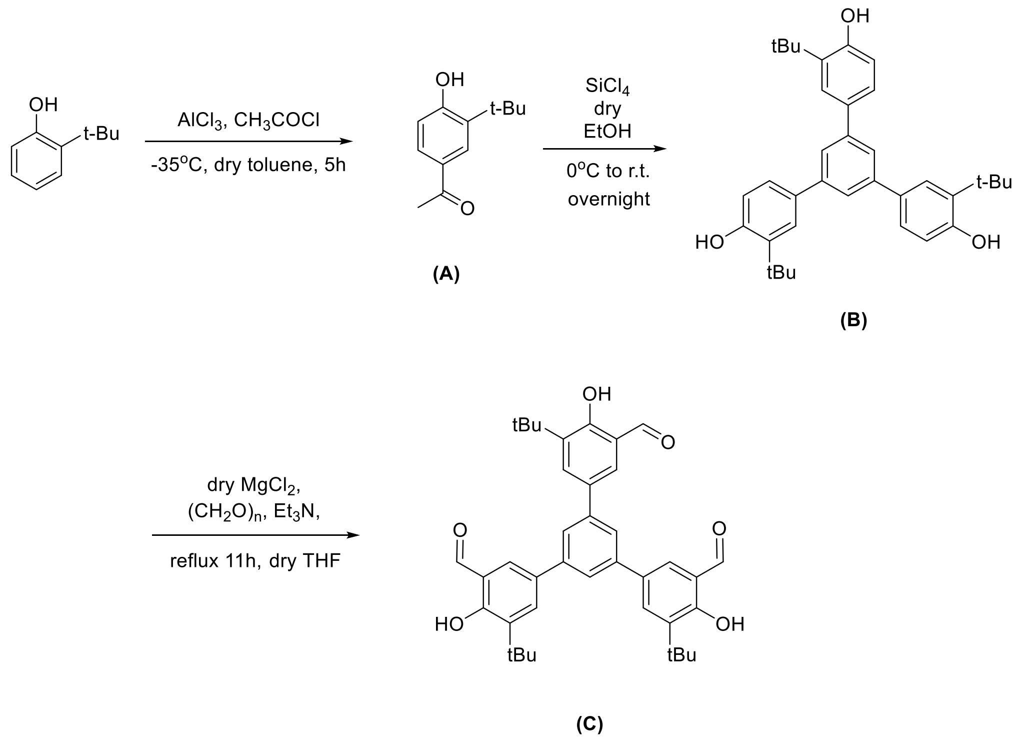

Scheme S1. Synthesis of compounds **(A)** – **(C)**

Synthesis of 3-tert-butyl-4-hydroxyacetophenone (A)

An oven dried 250 mL round-bottom flask was charged with anhydrous aluminium chloride (10.67g, 80 mmol, 1.2 equiv.) and dried toluene (200 mL). The yellow suspension was stirred and cooled to -35 °C and 2-tert-butylphenol (10.3 mL, 66.5 mmol, 1.0 equiv.) in 10 mL of dried toluene was added dropwise to the slurry and stirred for 1 h. To the pale yellow solution obtained, acetyl chloride (6.28 g, 80mmol, 1.2 equiv.) in 10 mL of dried toluene was added dropwise and stirred at this temperature for 5 h. After heating to room temperature, the mixture was poured into the 250 mL of stirred ice water. The organic phase was separated and the aqueous phase was extracted with 5 x 50 mL of ethyl acetate. The combined extracts were washed with brine (2 x 100 mL), dried over Na_2SO_4 and evaporated on a rotary evaporator. The crude product was triturated with ethyl acetate/hexane (1/30) to afford the pure product as a white powder (10.0 g, 78%).

^1H NMR (500 MHz, $CDCl_3$) δ: 7.96 (d, J = 2.2 Hz, 1H, Ph-*H*), 7.73 (dd, J = 8.3, 2.2 Hz, 1H, Ph-*H*), 6.79 (d, J = 8.3 Hz, 1H, Ph-*H*), 6.65 (s, 1H, O*H*), 2.57 (s, 3H, CH_3), 1.43 (s, 9H, $(CH_3)_3$).

Synthesis of 1,3,5-tris(3'-tert-butyl-4'-hydroxyphenyl)benzene (B)

An oven dried 150 mL round-bottom flask was charged with 3-tert-butyl-4-hydroxyacetophenone (2.4 g, 13.2 mmol) and dry ethanol (30 mL) and stirred at 0 ^{c}o. for 15 min. Silicon tetrachloride (7.6 mL, 66 mmol) was then added dropwise and stirred at this temperature for 30 min. The reaction mixture was warmed up slowly to room temperature and stirred overnight. The reaction was quenched with water and extracted with CH_2Cl_2 (3 x 50 mL). The organic layer was washed with water, dried over $MgSO_4$, and concentrated in vacuum. The residue was purified by silica gel column chromatography (CH_2Cl_2/hexane 1:1) to give target product as a white solid (1.40 g, 63%).

^1H NMR (500 MHz, $CDCl_3$) δ: 7.63 (s, 3H, Ph-*H*), 7.60 (d, *J* = 2.2 Hz, 3H, Ph-*H*), 7.41 (dd, *J* = 8.1, 2.2 Hz, 3H, Ph-*H*), 6.79 (d, *J* = 8.1 Hz, 3H, Ph-*H*), 4.96 (s, 3H, O*H*), 1.49 (s, 27H, $C(CH_3)_3$).

^{13}C NMR (125MHz, $CDCl_3$) δ: 154.08, 142.58, 136.58, 134.12, 126.59, 126.02, 124.30, 117.10, 34.88, 29.78.

Synthesis of 1,3,5-tris(3'-tert-butyl-4'-hydroxy-5'-formylphenyl)benzene (C)

An oven dried 250 mL round-bottom flask was charged with anhydrous magnesium dichloride (1.4 g, 14.7 mmol), solid paraformaldehyde (0.7 g, 22.6 mmol) and dry THF (50 mL). 2 mL of triethylamine was added via syringe dropwise and the resultant mixture was stirred at room temperature for 10 min. A solution of 1,3,5-tris(3-tert-butyl-4-hydroxyphenyl)benzene (1.24 g, 2.4 mmol) in 50 mL dry THF was added dropwise and the

reaction mixture was refluxed for 11 h. The reaction mixture was cooled to room temperature and 50 mL of ethyl acetate and 50 mL of 1N HCl was added. The resulting organic phase was separated and washed with 1 N HCl (2 x 100 mL) and water (3 x 100 mL), dried over $MgSO_4$, and concentrated in vacuum. The residue was purified by silica gel column chromatography (CH_2Cl_2/hexane 1:3) to give target product as a pale yellow solid (1.17 g, 82%).

^{1}H NMR (500 MHz, $CDCl_3$) δ: 11.86 (s, 3H, O*H*), 10.00 (s, 3H, C*H*O), 7.83 (d, *J* = 2.1 Hz, 3H, Ph-*H*), 7.69 (d, *J* = 2.3 Hz, 3H, Ph-*H*), 7.64 (s, 3H, Ph-*H*), 1.50 (s, 27H, C(CH_3)$_3$).

^{13}C NMR (125 MHz, $CDCl_3$) δ: 197.30, 161.13, 142.03, 139.30, 133.42, 132.33, 130.45, 124.54, 120.93, 35.27, 29.39.

IR (KBr): $\tilde{\upsilon}$ = 3281 (m), 3037 (m), 2954 (vs), 2916 (vs), 2867 (s), 1651 (vs), 1614 (vs), 1592 (vs), 1471 (vs), 1394 (vs), 1318 (vs), 1236 (s), 1166 (s), 1099 (m), 1026 (m), 972 (m), 870 (s), 771 (s), 720 (s), 629 (m)

Resolution of (*S*,*S*)-*trans*-1,2-diaminocyclohexane (D)

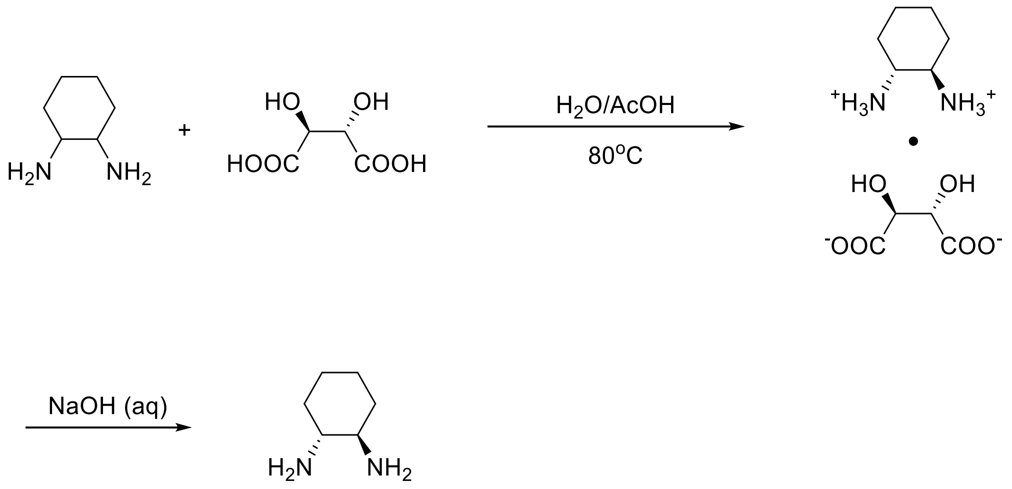

(D)

Scheme S2. Resolution of (*S*,*S*)-*trans*-1,2-diaminocyclohexane **(D)**

(+)-(*S*,*S*)-1,2-diaminocyclohexane.2L-tartrate. In a 50 mL round-bottomed flask, (+)-L-tartaric acid (6.0 g, 40 mmol) and distilled water (17 mL) were added and stirred until all the acid had dissolved. Commercially available 1,2-diaminocyclohexane (mixture of *cis* and *trans* isomers, 10 mL, 72 mmol) and glacial acetic acid (4.6 mL, 80 mmol) were then added and the mixture was stirred for 2 h at room temperature and then placed in an ice bath for another 2 h. The precipitate was filtered and washed with distilled water and methanol. The filtrate was then stirred and heated to 80 °C. (+)-L-tartaric acid (15.0 g, 100 mmol) was again added to the filtrate and the solution was stirred overnight at room temperature and then cooled in an ice bath. The precipitate was filtered, washed with cold distilled water (5 mL) and methanol (3 x 5 mL), and dried in vacuo to give (+)-(*S*,*S*)-1,2-diaminocyclohexane.2L-tartrate as a white solid (10.0 g).

$[\alpha]_D^{20}$ = +24.1 (*c* 1, H_2O), $[\alpha]_D^{20}$ = +25.4 (*c* 1, H_2O) (literature value[2])

(*S,S*)-*trans*-1,2-diaminocyclohexane (D). (+)-(S,S)-1,2-diaminocyclohexane.2L-tartrate (10.0 g, 24mmol) was dissolved in aqueous sodium hydroxide (4 M, 100 mL) and loaded into a 500 mL separatory funnel. The mixture was extracted with dichloromethane (6 x 50 mL) and the combined organic extracts were washed with brine (100 mL), dried over anhydrous Na_2SO_4, and concentrated under reduced pressure to give pale yellow oil. The oil was dissolved in anhydrous hexane (150 mL), filtered through a medium porosity fritted glass disc filter and concentrated under reduced pressure. The resultant oil was dried under high vacuum for 10 - 15 minutes to give (S,*S*)-1,2-diaminocyclohexane as a white solid (1.7 g, 63%).

$[\alpha]_D^{20}$ = +17.2 (*c* 1, 1M HCl), $[\alpha]_D^{20}$ = +17.0 (*c* 1, 1M HCl) (literature value[2])

Synthesis of salen@cage

In an oven dried round-bottomed flask, compounds (**C**) (436 mg, 0.72 mmol, 2 equiv.) and (**D**) (136 mg, 1.08 mmol, 3.3 equiv.) were dissolved in dry DMF (100 mL) and a solution of trifluoroacetic acid (TFA) in dry DMF (2 mol%, 0.05 M, 200 μL) was added. The solution turned cloudy upon addition of TFA and the mixture was stirred at 120°C for 5 days. The resultant yellow suspension was allowed to cool to room temperature, filtered and the solid obtained was washed with dry DMF (3 x 25 mL), diethyl ether (3 x 25 mL) and hexane (3 x 25 mL), and dried in vacuo for 24 h to obtain salen@cage as a bright yellow solid (300 mg, 58%).

^{13}C CP MAS NMR: δ: 162.6, 157.2, 140.0, 134.9, 124.7, 116.1, 70.7, 61.6, 31.8, 26.7, 21.8.

IR (KBr): $\tilde{\upsilon}$ = 2949 (s), 2863 (s), 1630 (vs), 1591 (s), 1467 (s), 1443 (s), 1392 (m), 1315 (w), 1268 (m), 1217 (w), 1169 (m), 1096 (w), 864 (m), 774 (m), 716 (w), 633 (w).

MS (MALDI-TOF, dithranol): *m/z*: 1448.0.

MS (ESI): m/z (%) = 1447.83 $[M]^+$ (100)

HRMS (ESI) m/z calcd for $C_{96}H_{115}N_6O_6$, $[M+H]^+$ 1448.8912, found: 1448.8977

EA: Calcd. $C_{96}H_{114}N_6O_6.H_2O$: C 78.65, H 7.98, N 5.73 %. Found: C 78.98, H 7.64, N 5.67 %.

Synthesis of Co(II)@cage, Co(III)@cage and Al(III)@cage

Co(II)@cage. salen@cage (0.3 mmol, 435 mg, 1 equiv.) and dry CH_2Cl_2 (50 mL) were added into an oven dried round-bottomed flask and stirred vigorously. $Co(OAc)_2.4H_2O$ (1.08 mmol, 270 mg, 3.6 equiv.) was dissolved in dry CH_3OH (50 mL) and added via syringe into the mixture. The brick-red suspension was heated to 70 °C and stirred for 3 days. After cooling to room temperature, the dark red solid was filtered and washed with CH_3OH to remove any residues of the metal salt. Further purification of the compound was done by Soxhlet extraction with a 1:1 volume ratio of dry CH_2Cl_2 and dry CH_3OH for 22 h. The product was dried in vacuo for 24 h and isolated as a dark red powder (412 mg, 85%).

IR (KBr): $\tilde{\upsilon}$ = 2944 (s), 2863 (s), 1630 (shoulder), 1607 (vs), 1587 (vs), 1527 (s), 1426 (s), 1386 (m), 1337 (m), 1320 (m), 1240 (w), 1168 (m), 1094 (w), 1027 (w), 939 (w), 861 (m), 816 (w), 787 (m), 725 (w), 646 (m)

EA: Calcd. $C_{96}H_{108}Co_3N_6O_6.5H_2O$: C 67.48, H 6.96, N 4.92, Co 10.35 %. Found: C 67.64, H 6.37, N 5.24, Co 7.44 %. Low Co % was due to incomplete metalation of Co onto salen@cage (see Table S1).

Co(III)@cage. Co(II)@cage (0.2 mmol, 324 mg, 1 equiv.) and dry CH_2Cl_2 (6 mL) were added into an oven dried Schlenk flask and stirred vigorously under an oxygen atmosphere. Glacial acetic acid (0.35 mL, ~30 equiv.) dissolved in dry CH_2Cl_2 (6 mL) was added slowly to the mixture and stirred under an oxygen atmosphere overnight. The colour of the mixture turned from dark red to dark brown which indicated that the Co(II) metal centres have been

successfully oxidized to Co(III) as these colour changes are associated with Co(II) salen and Co(II) salen systems.[3] The brown solid was filtered and washed copiously with CH_2Cl_2 to remove the excess acetic acid. The product was dried in vacuo for 24 h and isolated as a brown powder (330 mg, 92%).

^{13}C CP MAS NMR: δ: 163.35, 143.09, 127.46, 72.33, 29.45.

IR (KBr): $\tilde{\upsilon}$ = 2947 (s), 2864 (s), 1630 (shoulder), 1609 (vs), 1589 (vs), 1528 (s), 1428 (s), 1392 (m), 1337 (m), 1323 (m), 1242 (w), 1167 (m), 1096 (w), 1028 (w), 949 (w), 862 (m), 816 (w), 783(m), 725 (w), 647 (m)

EA: Calcd. $C_{102}H_{125}Co_3N_6O_{16}.5H_2O$: C 64.96, H 6.79, N 4.46, Co 9.37 %. Found: C 64.91, H 6.78, N 4.66, Co 6.73 %. Low Co % was due to incomplete metalation of Co onto salen@cage (see Table S1).

Al(III)@cage. salen@cage (0.3 mmol, 435 mg, 1 equiv.), $Al(OEt)_3$ (1.08 mmol, 175 mg, 3.6 equiv.) and dry toluene (120 mL) were added into an oven dried round-bottomed flask and stirred vigorously. The yellow suspension was heated to 110 °C and stirred for 3 days. After cooling to room temperature, the yellow solid was filtered and washed with toluene to remove any residues of the metal salt. Further purification of the compound was done by Soxhlet extraction with toluene for 24 h and ethanol for 24 h. The product was dried in vacuo for 24 h and isolated as a yellow powder (440 mg, 89%).

^{13}C CP MAS NMR: δ: 167.72, 162.42, 144.37, 130.03, 120.77, 65.08, 36.24, 30.71, 26.12.

IR (KBr): $\tilde{\upsilon}$ = 2948 (s), 2863 (s), 1627 (vs), 1590 (vs), 1543 (s), 1469(s), 1437 (s), 1391 (m), 1360 (m), 1315 (w), 1267 (w), 1243 (w), 1200 (w), 1171 (m), 1096 (w), 1028 (w), 982 (w), 930 (w), 864 (m), 788(w), 718 (w), 644 (m)

EA: Calcd. $C_{102}H_{123}Al_3N_6O_9.2H_2O$: C 72.32, H 7.56, N 4.96, Al 4.78 %. Found: C 72.63, H 7.77, N 5.28, Al 5.77 %. High Al % might be due to an excess of insoluble $Al(OEt)_3$ or $Al(OH)_3$ present.

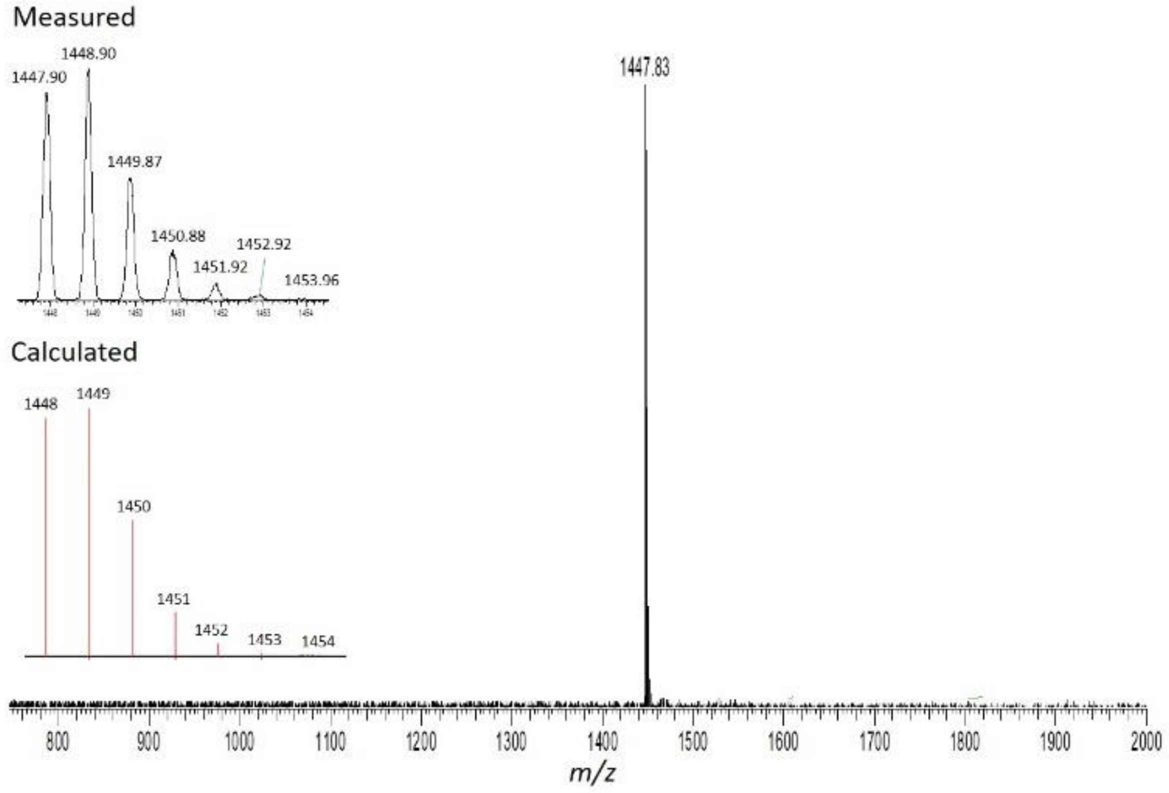

Figure S1. ESI-MS of salen@cage. The insets show the measured isotopic pattern (top) by ESI-MS and the calculated isotopic pattern (bottom).

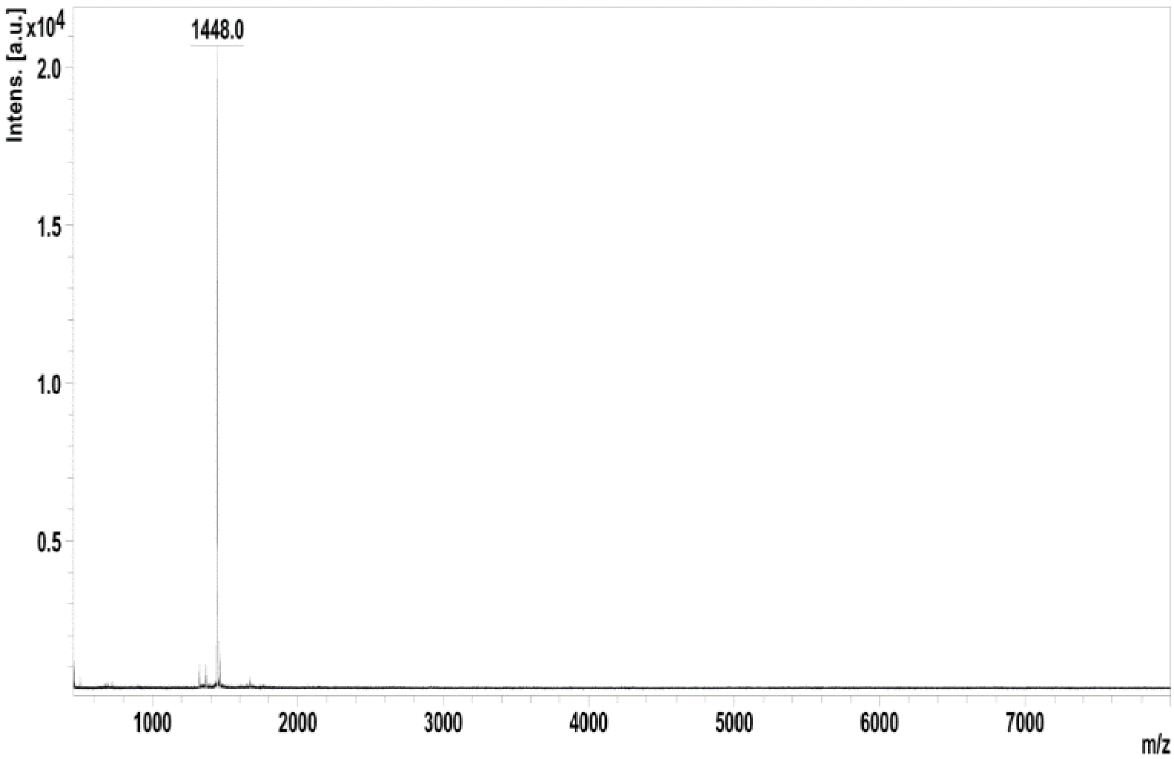

Figure S2. MALDI-TOF MS of salen@cage. In order to exclude the formation of smaller (e.g. [2+1] or [2+2]) or larger (e.g. [4+6] or up to [10+15]) condensation cages, a mass spectrum of m/z 750 – 8000 was recorded by MALDI-TOF MS. No further signals other than salen@cage (m/z 1448.0) were detected (Figure 2).

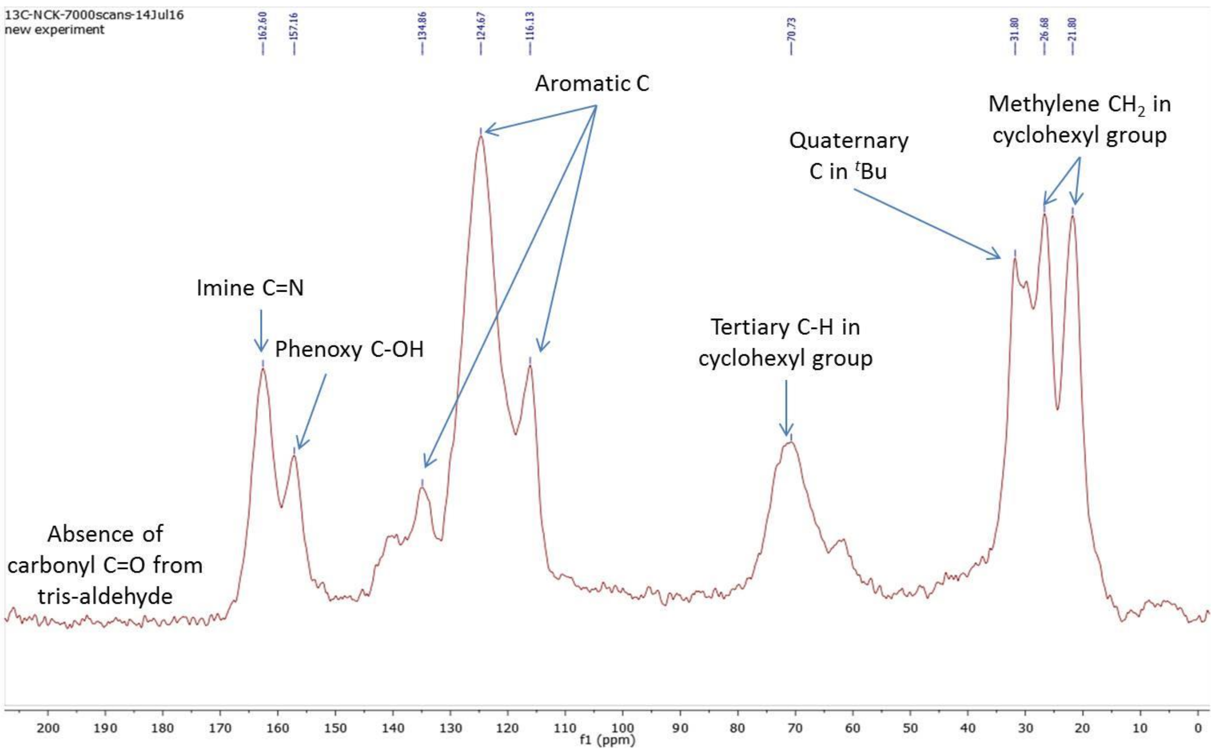

Figure S3. ^{13}C CP MAS NMR spectrum of salen@cage. The most downfield signal at δ = 162.6 ppm corresponds to the imine C=N carbon nuclei, comparable to other imine C=N carbon nuclei in molecular cages.[4] The absence of obvious signals in the region δ = 180 – 220 ppm, which correspond to the carbonyl C=O for aldehydes, also indicated that the tris-aldehyde starting material was clearly absent from salen@cage.

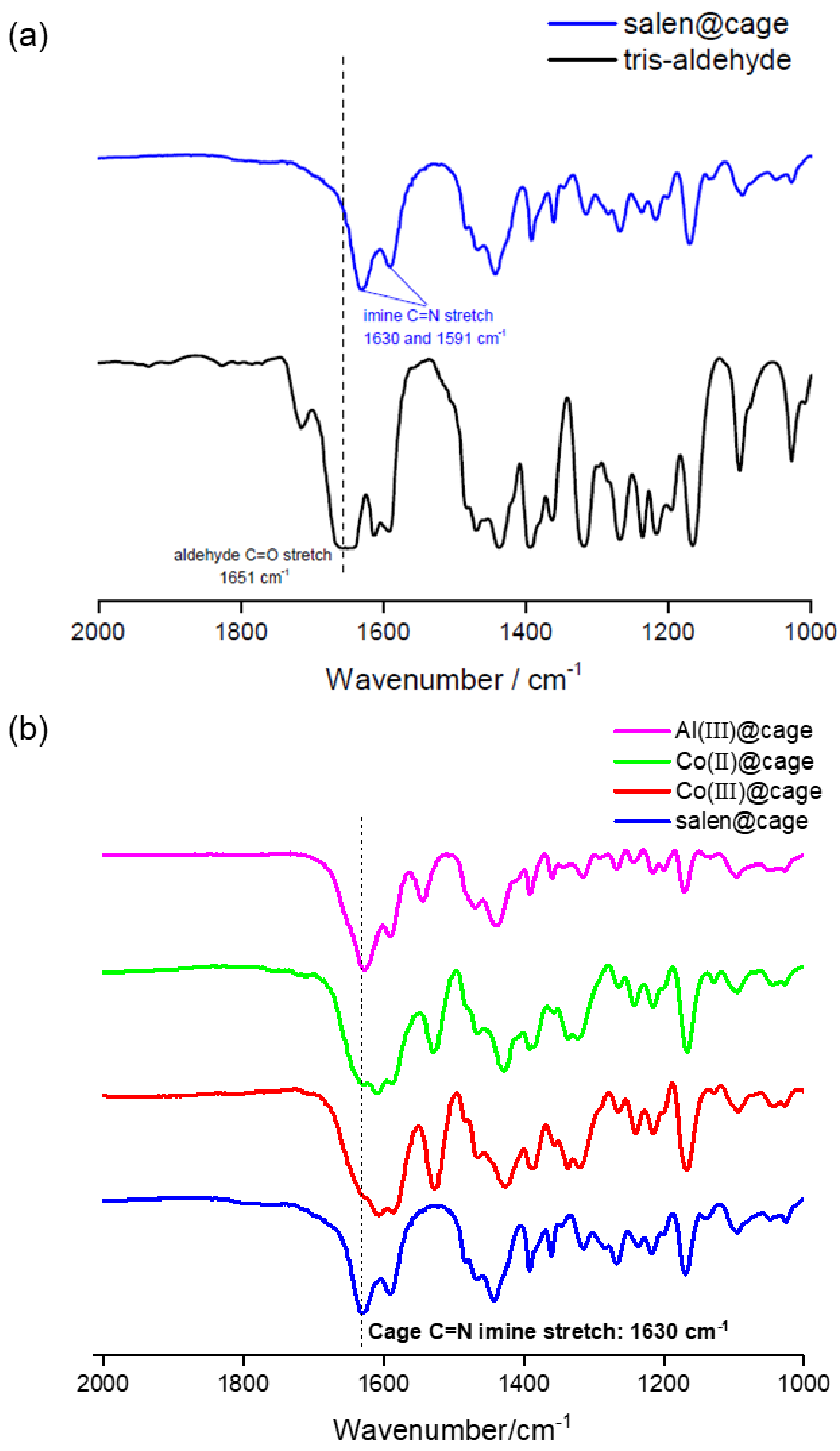

Figure S4a. IR spectra of tris-aldehyde (**C**) and salen@cage. The spectra were recorded using KBr pellets. strong C=N imine stretching bands at $\tilde{\upsilon}$ = 1630 and 1591 cm^{-1}. The C=O

stretching band of the tris-aldehyde at ~ 1651 cm^{-1} was not detected which further showed that no starting material was left in salen@cage. **Figure S4b.** IR spectra of the cage complexes. The spectra were recorded using KBr pellets. Upon metalation of the salen@cage with Al or Co, the C=N imine stretch at 1630 cm^{-1} shifts to lower frequencies to indicate successful metalation.

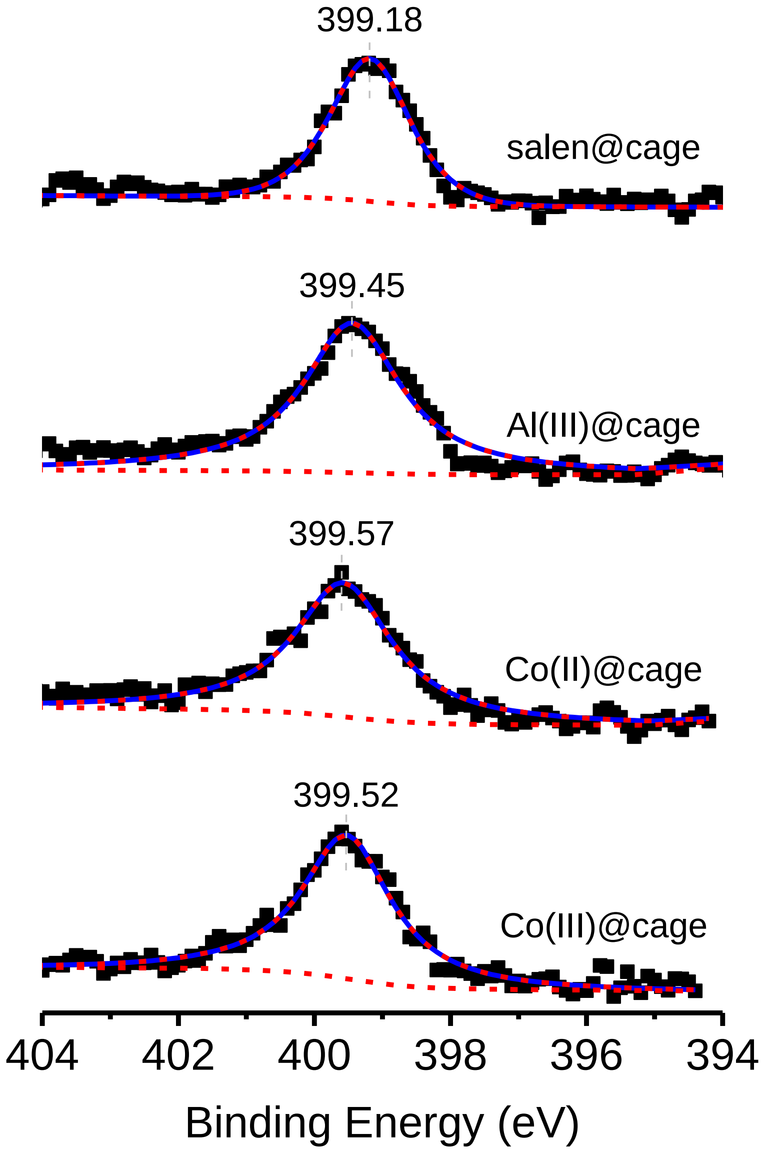

Figure S5. N 1s XPS spectra of salen@cage, Al(III)@cage, Co(II)@cage and Co(III)@cage.

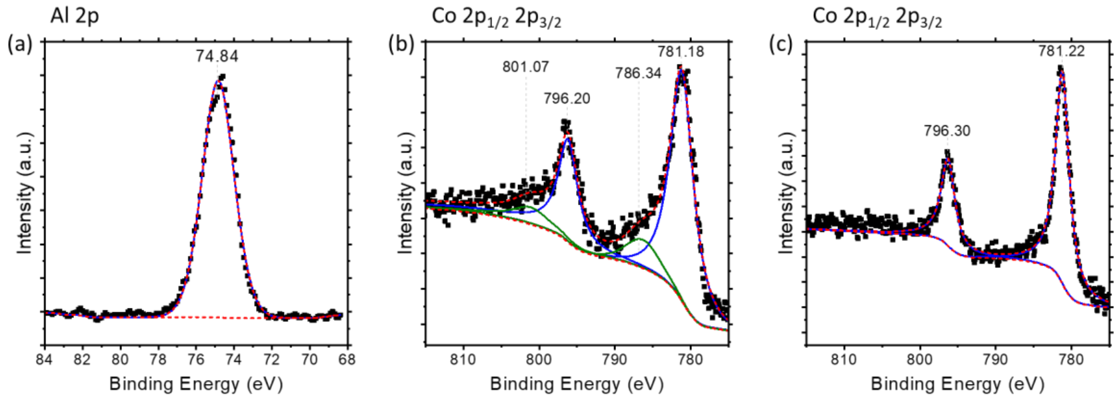

Figure S6. (a) Al 2p XPS spectrum of Al(III)@cage corresponding to Al in the +3 oxidation state, (b) Co 2p XPS spectrum of Co(II)@cage corresponding to Co in the +2 oxidation state due to the presence of satellite features,[5] and (c) Co 2p XPS spectrum of Co(III)@cage corresponding to Co in the +3 oxidation state due to the absence of satellite features and slightly higher binding energy.[6]

Table S1. Elemental analysis of Co(II)@cage, Co(III)@cage and Al(III)@cage

	C (%)	H (%)	N (%)	N (mmol g^{-1})	Metal (%)	Metal (mmol g^{-1})	N/Metal	Metal Load (%)
Co(II)@cage	67.64	6.37	5.24	3.74	7.44	1.26	2.97	67
Co(III)@cage	64.91	6.78	4.66	3.33	6.73	1.14	2.92	68
Al(III)@cage	72.63	7.77	5.28	3.77	5.77	2.14	1.76	114

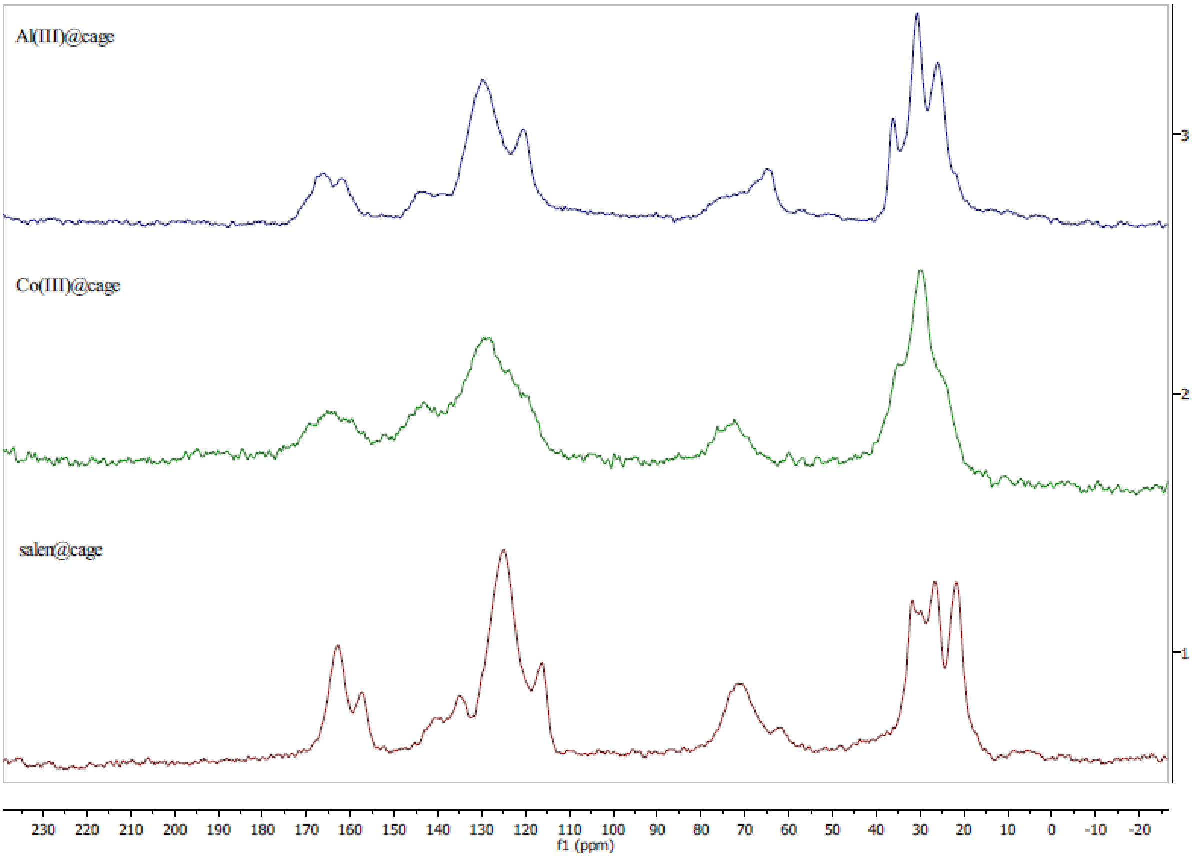

Figure S7. ^{13}C CP MAS NMR spectra of salen@cage, Co(III)@cage and Al(III)@cage.

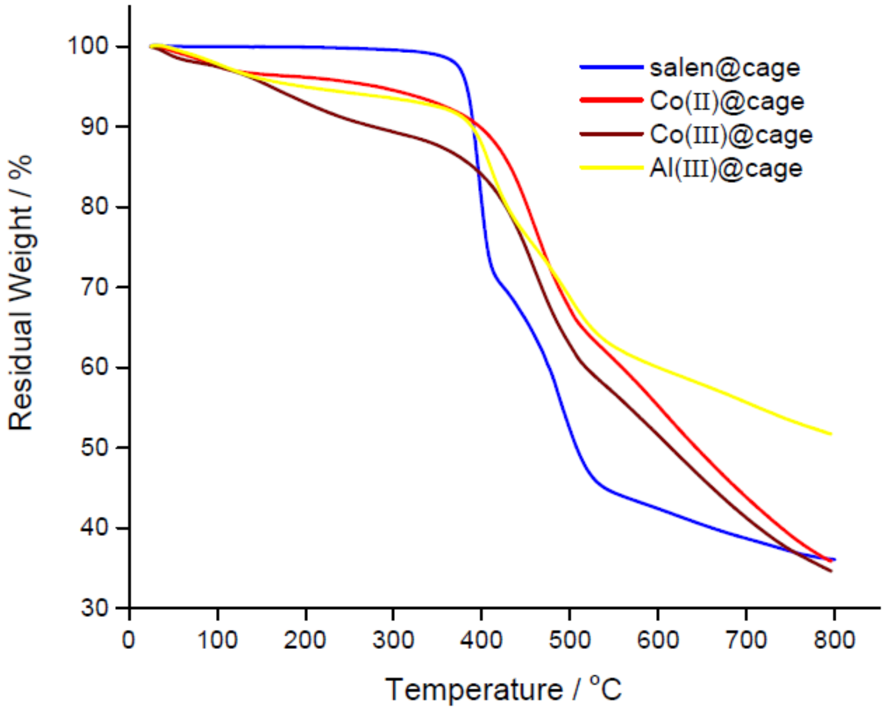

Figure S8. Thermogravimetric analysis (TGA) of the cage complexes under N_2.

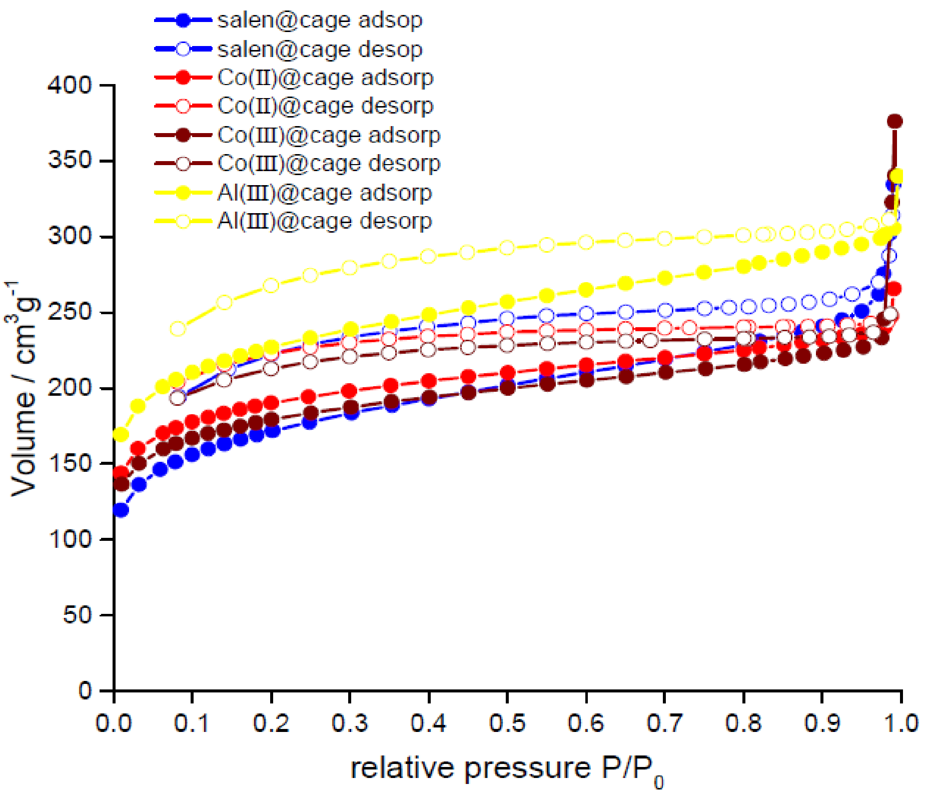

Figure S9. N_2 adsorption and desorption isotherms for the synthesized cages at 77K.

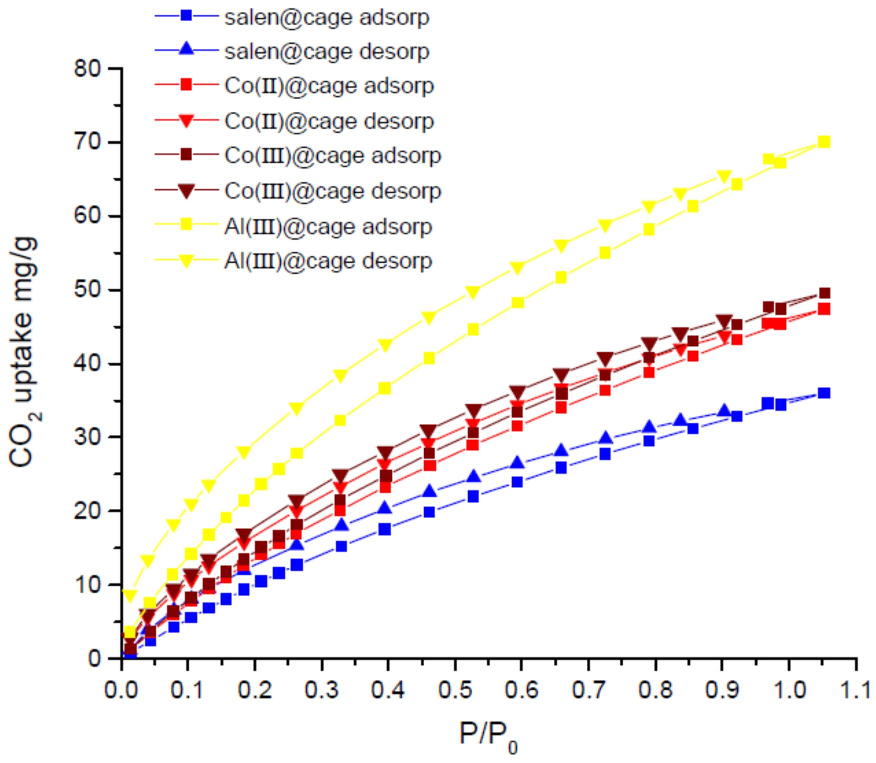

Figure S10. CO_2 adsorption and desorption isotherms for the cage complexes at 298K.

Table S2. Gas sorption data for the cage complexes

Cage complex	SA_{BET} $(m^2g^{-1})^{a,b}$	SA_{micro} $(m^2g^{-1})^{c}$	V_{total} $(cm^3g^{-1})^{d}$	V_{micro} $(cm^3g^{-1})^{e}$	CO_2 uptake $(mg\ g^{-1})^{f}$
salen@cage	621	276	0.517	0.114	35.8
Co(II)@cage	635	388	0.376	0.161	47.1
Co(III)@cage	610	363	0.543	0.150	49.2
Al(III)@cage	771	430	0.491	0.179	70.4

[a]SA = surface area, [b]Brunauer Emmett Teller (BET) surface area calculated over (P/P_0) 0.01–0.14. [c]Micropore surface area calculated from the N_2 adsorption isotherm using the t-plot method. [d]Total pore volume calculated at $P/P_0 = 0.99$. [e]Micropore volume calculated using the t-plot method. [f]Volumetric CO_2 adsorption–desorption isotherms measured for the cage complexes at 298 K.

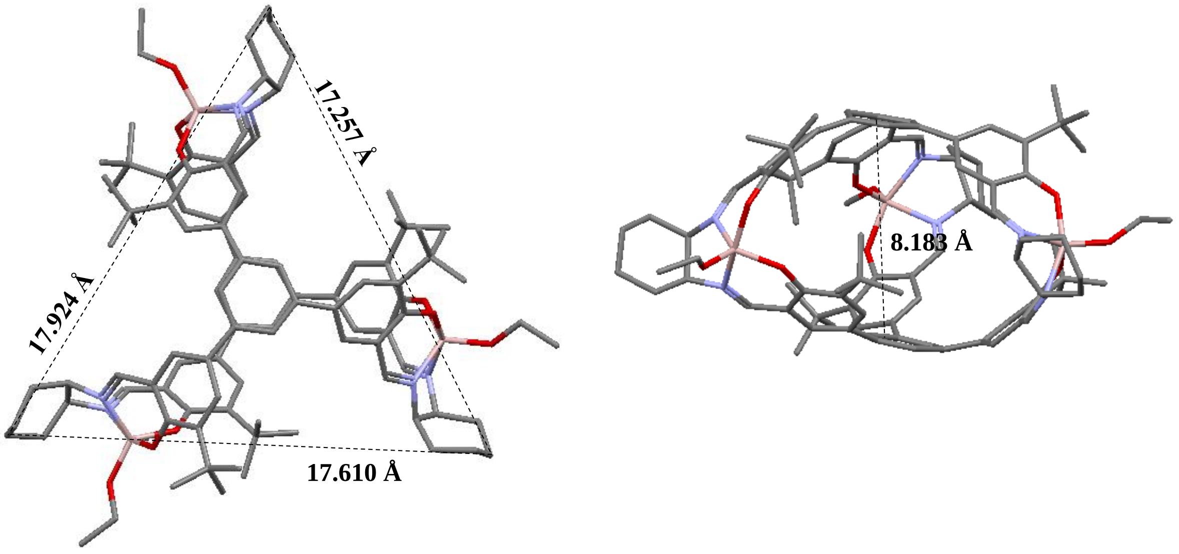

Figure S11. Molecular dimensions of Al(III)@cage based on DFT B3LYP/6-31G (Refer to Table S4).

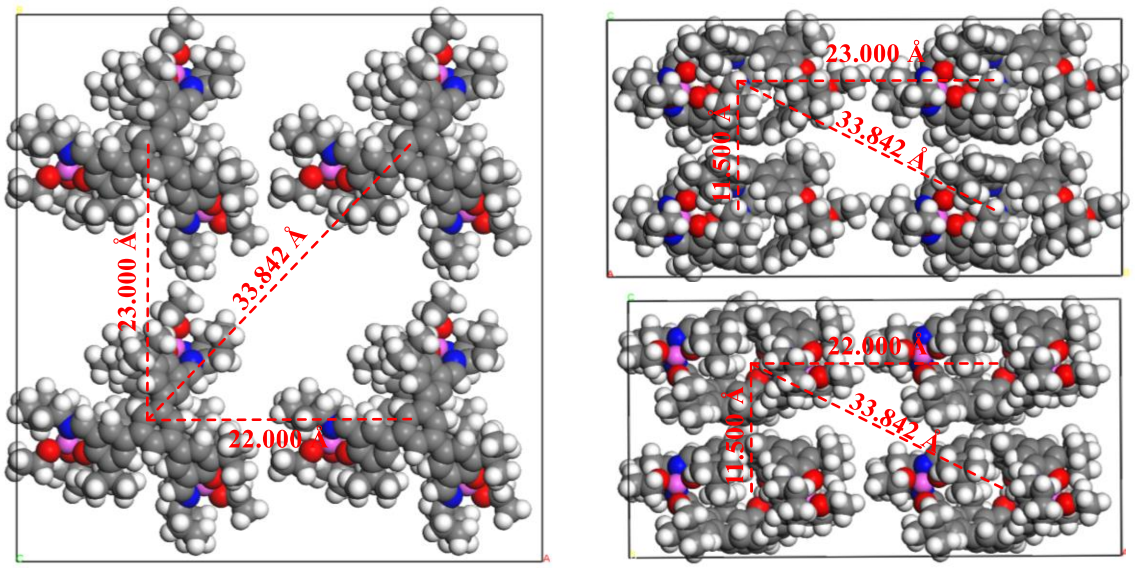

Figure S12. Simulated molecular packing of Al(III)@cage. Packing of the cage (supercell, a = 44, b = 46, c = 23 angstroms) was built in Materials Studio and using G09 DFT optimized single cage geometry.

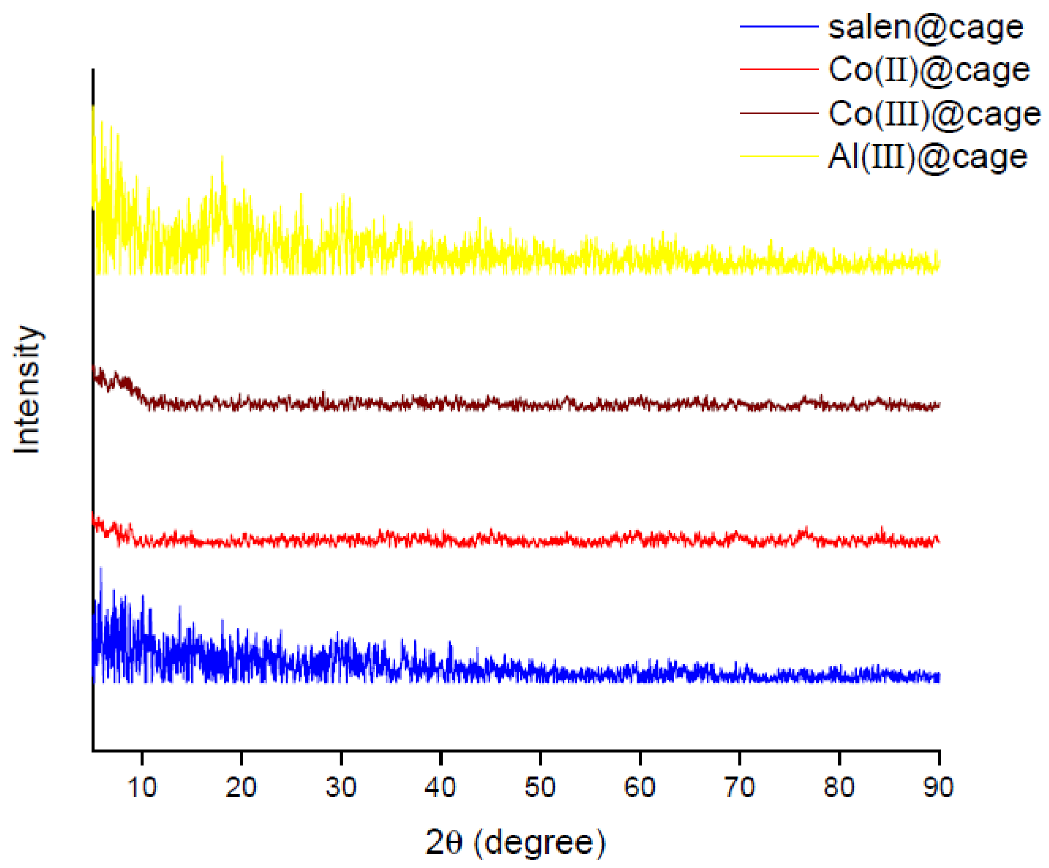

Figure S13. PXRD of the cage complexes

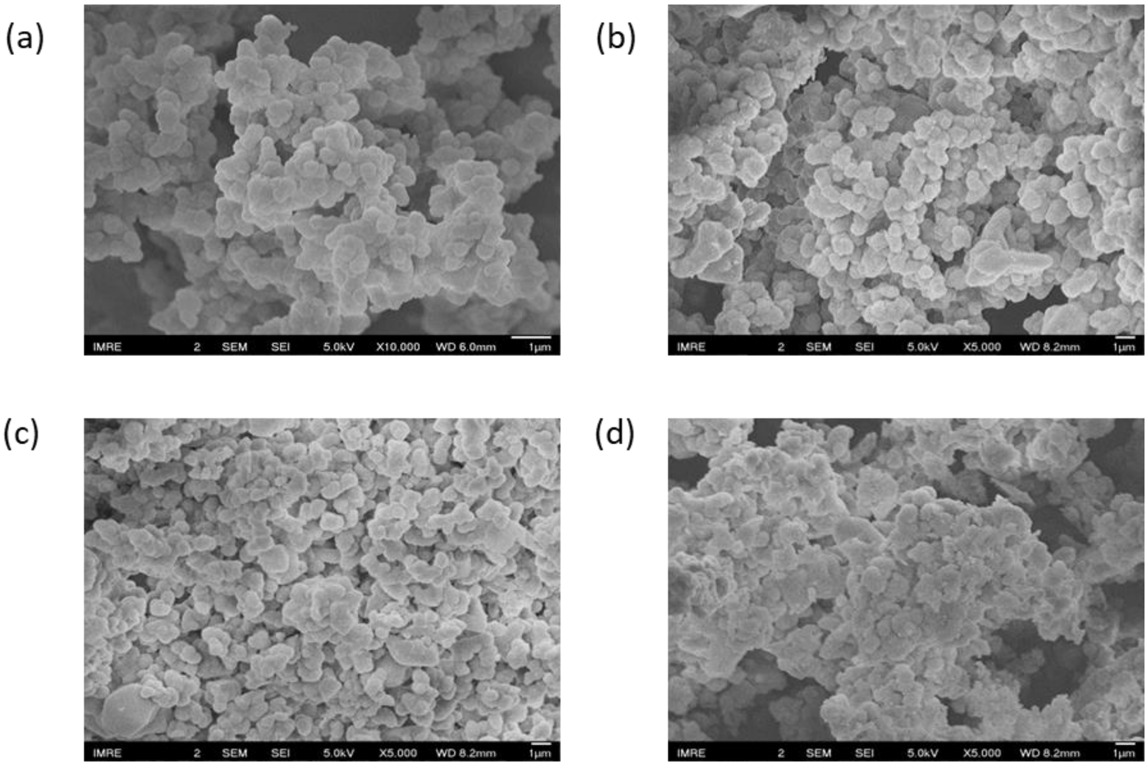

Figure S14. SEM of (a) salen@cage at 10,000x, (b) Co(II)@cage at 5,000x, (c) Co(III)@cage at 5,000x, (d) Al(III)@cage at 5,000x.

S3. Catalytic cycloaddition of CO_2 with epoxides and catalyst recycling

General procedure for cycloaddition of CO_2 with styrene oxide at room temperature and pressure.

The reaction was typically carried out in an oven-dried 25 mL Schlenk tube using styrene oxide (5 mmol, 0.60 g) in solvent free environment under a CO_2 balloon. The catalyst and tetra-*n*-tertbutylammonium bromide (TBAB) was first added into the Schlenk tube and the tube was evacuated and backfilled with CO_2 three times. Then styrene oxide was added and the reaction was stirred at the required temperature for a certain amount of time. After the reaction was complete, a small amount of the reaction mixture was sampled, diluted with $CDCl_3$ and filtered through a small pad of Celite to remove the solid catalyst. The solution was then analysed by ^1H NMR spectroscopy to calculate the conversion of styrene oxide to styrene carbonate.

General procedure for cycloaddition of CO_2 with epoxide substrates at room temperature and pressure using Al(III)@cage as catalyst.

The reaction was typically carried out in an oven-dried a 10mL Biotage Microwave Reaction V-shaped vial with the epoxide (1 mmol) in solvent free environment under a CO_2 balloon. The catalyst and tetra-*n*-tertbutylammonium bromide (TBAB) was first added into the vial and sealed with a rubber septum. The vial was evacuated and purged with CO_2 three times. Then 1 mmol of the epoxide was transferred into the vial via micro-syringe and the reaction was stirred at the room temperature for 48 h. The reaction mixture was diluted with methylene chloride and filtered through a short pad of silica to remove the catalyst and TBAB. The pure cyclic carbonates were obtained via flash chromatography using hexane:ethyl acetate (9:1 to 3:1).

Table S3. Screening of TBAB loading for Cycloaddition of CO_2 with Styrene Oxide to produce Styrene Carbonate at 25°C[a]

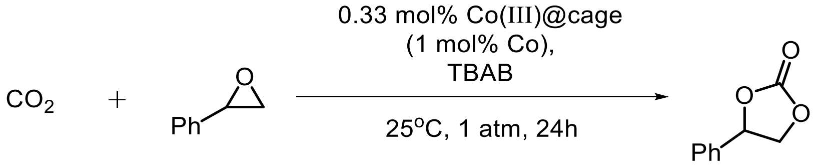

Entry	TBAB loading / mol%	Conversion / %[b]
1	NIL	0
2	1	67
3	2	84
4	5	95
5	10	100

[a]Typical reaction conditions: 5.0 mmol styrene oxide, TBAB and 0.33 mol% Co(III)@cage (1 mol% Co) under 1 atm CO_2 balloon at 25°C for 24 h. [b]Conversion calculated from ^1H NMR spectra.

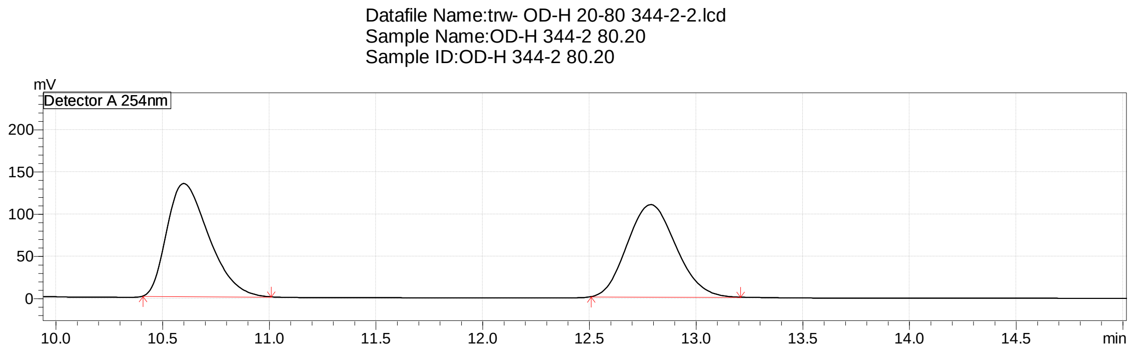

Figure S15. HPLC trace of styrene carbonate after cycloaddition of CO_2 to styrene oxide. OD-H, 80:20 hexane:*i*PrOH. Area percentage ratio: 51.3:48.7, ee = 2.6%. Reaction conditions: 0.33 mol% Al(III)@cage, 1 mol% TBAB, 10°C, 48 h, 1 atm CO_2.

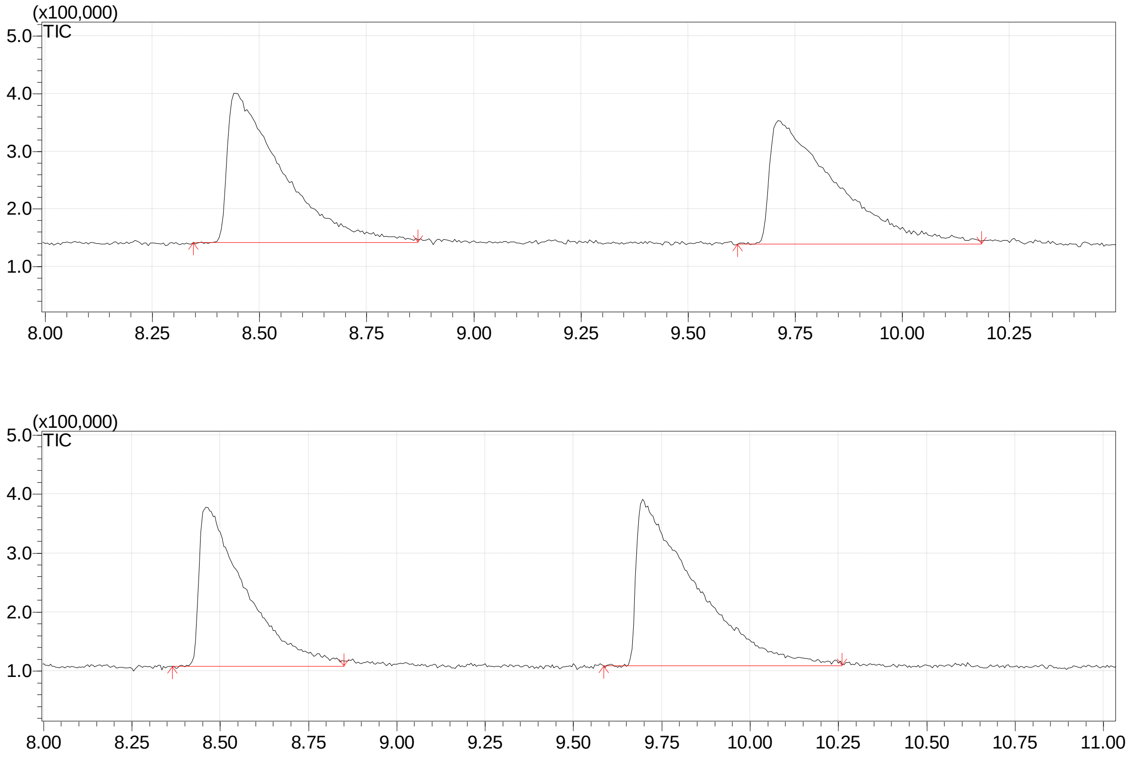

Figure S16. GC trace of racemic propylene carbonate (top). GC trace of propylene carbonate after cycloaddition of CO_2 to propylene oxide (bottom). Astec CHIRALDEX BTA column, 160°C isothermal, t_R(minor) = 8.46 min, t_R(major) = 9.70 min. Area percentage ratio: 43.2:56.8, ee = 13.6%. Reaction conditions: 0.33 mol% Al(III)@cage, 1 mol% TBAB, 10°C, 48 h, 1 atm CO_2.

Recycling experiments

For catalyst recycling experiments, the catalyst was recycled by filtration, washed with a copious amount of CH_2Cl_2 and then dried under vacuum overnight. The recycled catalyst was then reused for the next run without further purification.

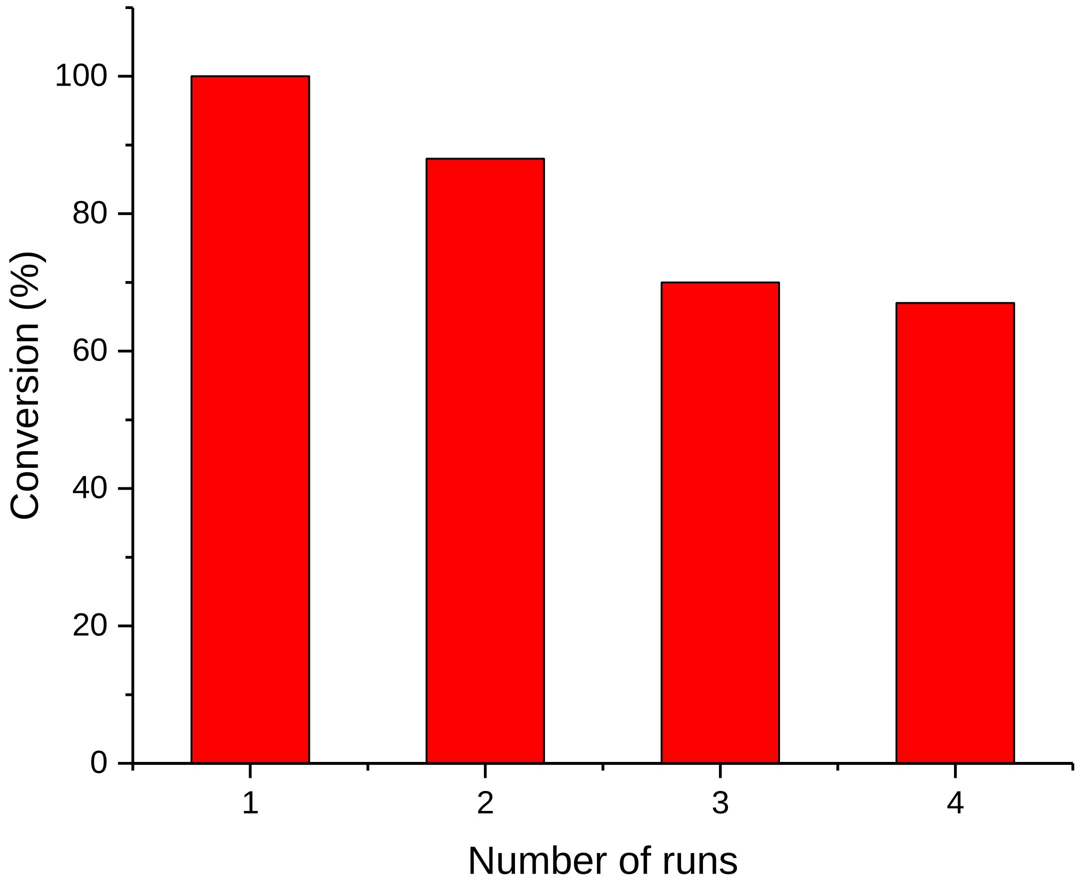

Figure S17. Recyclability of Co(III)@cage. Typical reaction condition for Co(III)@cage: 5.0 mmol styrene oxide, 10 mol% TBAB, 0.17 mol% Co(III)@cage, under 1 atm balloon at 25°C for 24 h. Conversions were not shown for the 5th run as insufficient catalyst could recovered after the 4th run.

S4. Mechanism of cycloaddition of styrene oxide with CO_2

The proposed mechanism of the cycloaddition of styrene oxide with CO_2 catalysed by Co(III)@cage or Al(III)@cage is shown in **Fig 4.14** below. The active catalytic centre is the Co(III) or Al(III) salen complex within the cage framework. Firstly, the metal-salen acts as a Lewis acid to coordinate onto styrene oxide to activate the ring for nucleophilic attack from Br^- from TBAB. Subsequently, CO_2 insertion occurs followed by the leaving of the nucleophile (Br^-) and desorption of the product styrene oxide from the catalytic centre

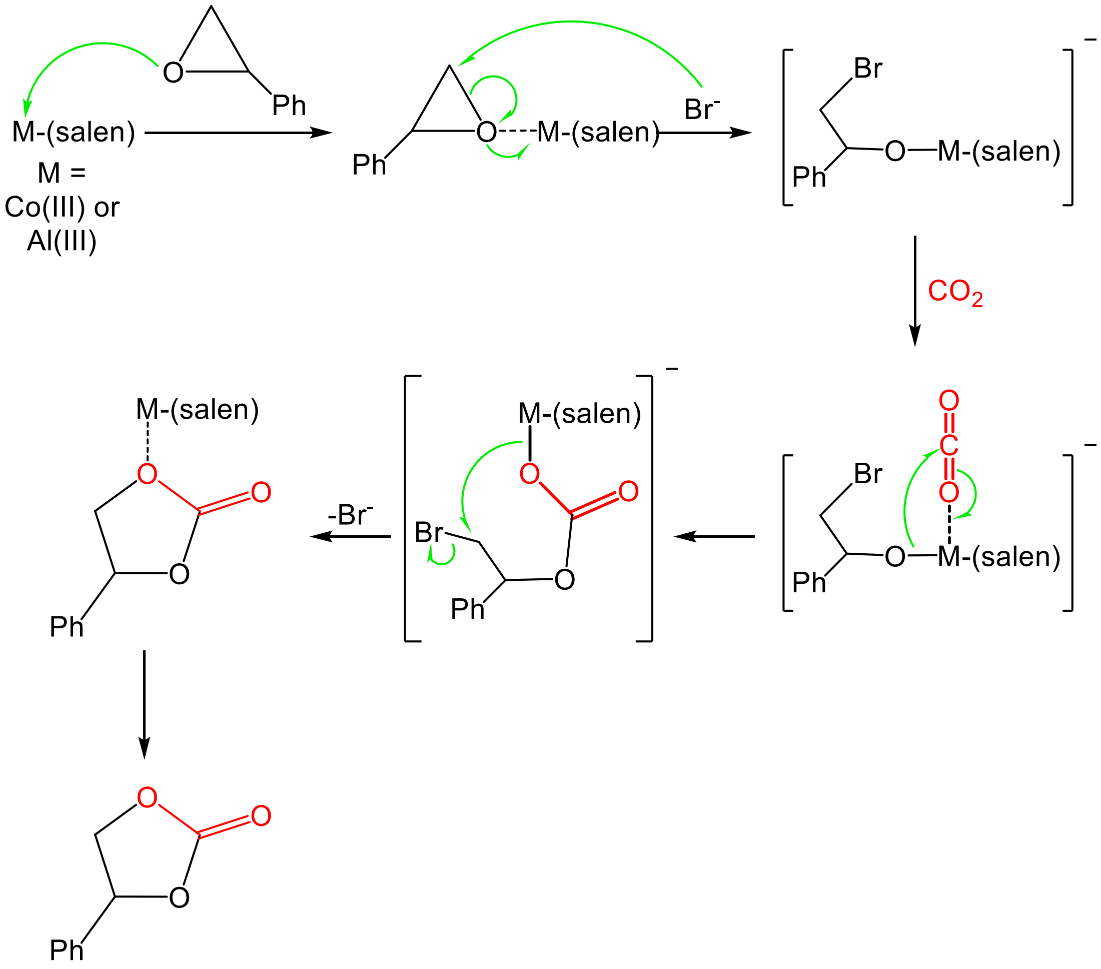

Figure S18. Proposed mechanism of cycloaddition of styrene oxide with CO_2 catalyzed by Co(III)@cage and Al(III)@cage.

S5. NMR spectra of cyclic carbonates

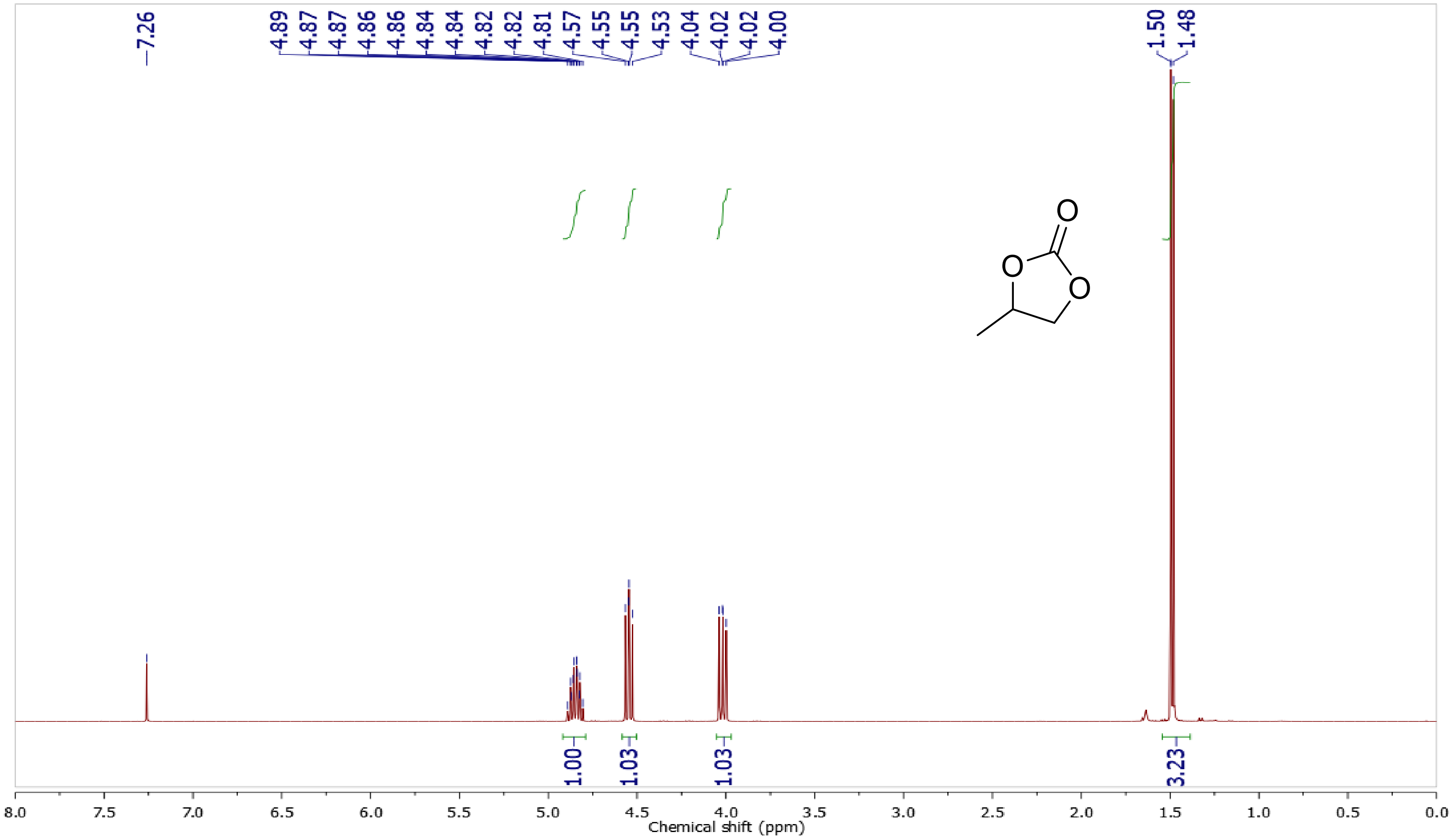

Figure S19. ^{1}H NMR of propylene carbonate in $CDCl_3$.

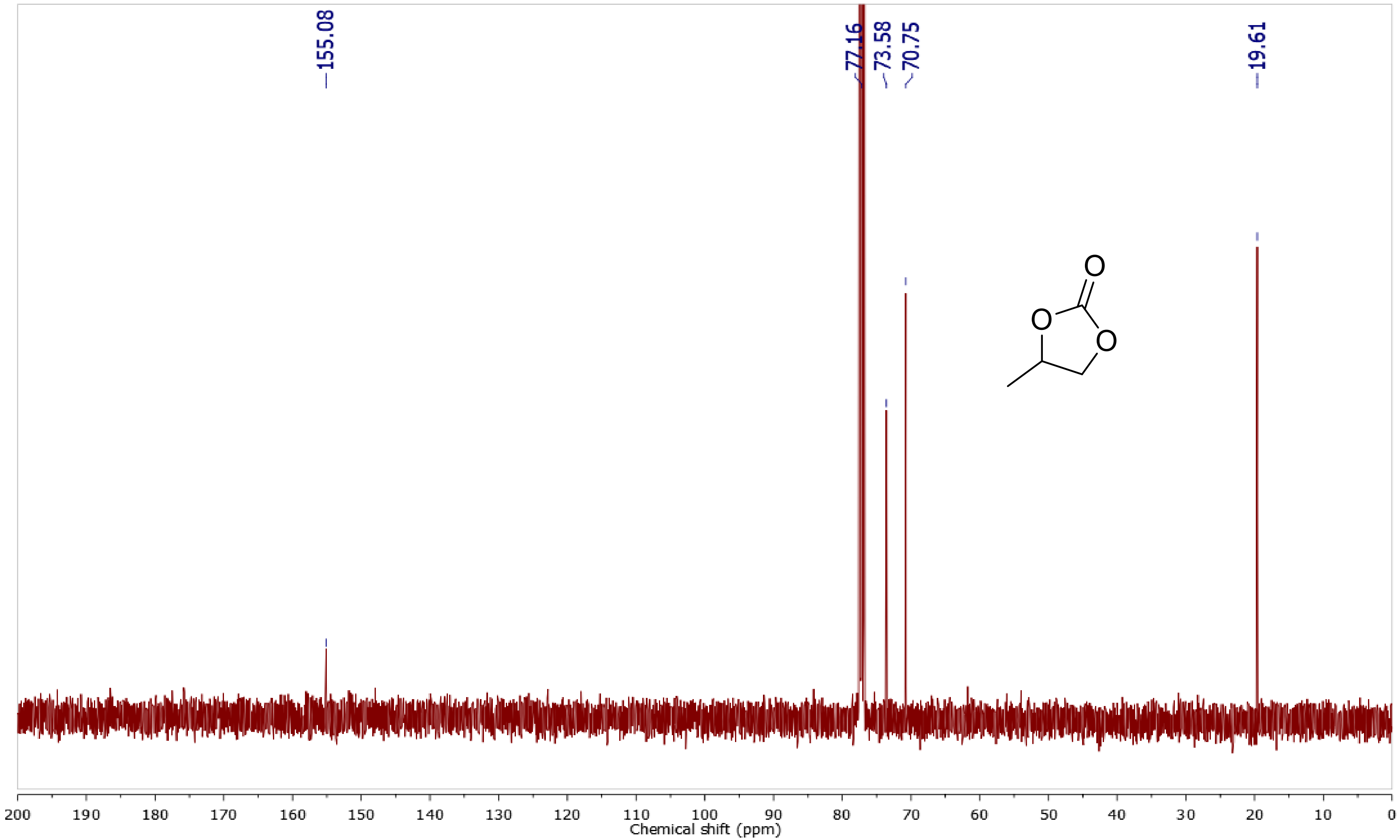

Figure S20. ^{13}C NMR of propylene carbonate in $CDCl_3$.

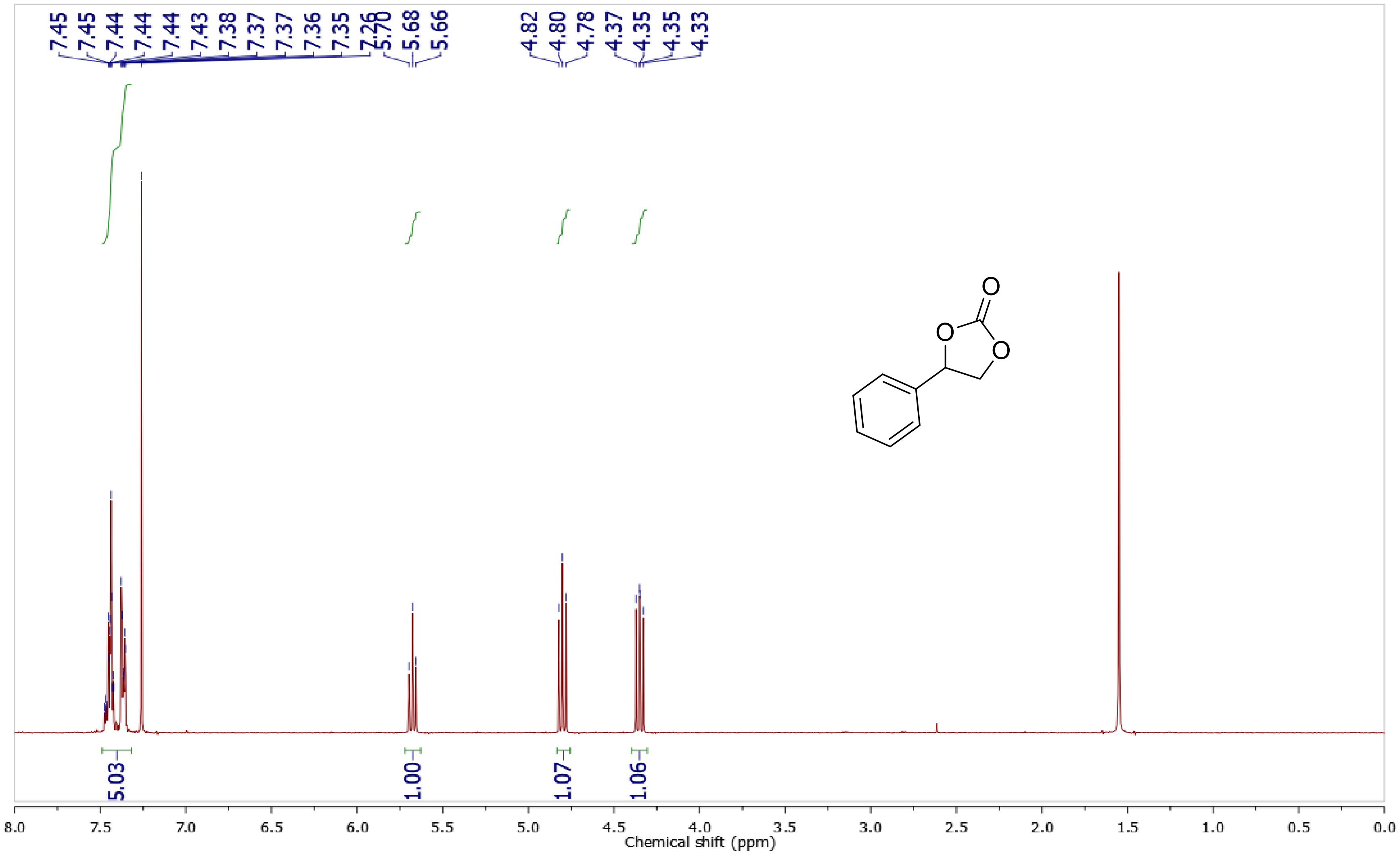

Figure S21. ^{1}H NMR of styrene carbonate in $CDCl_3$.

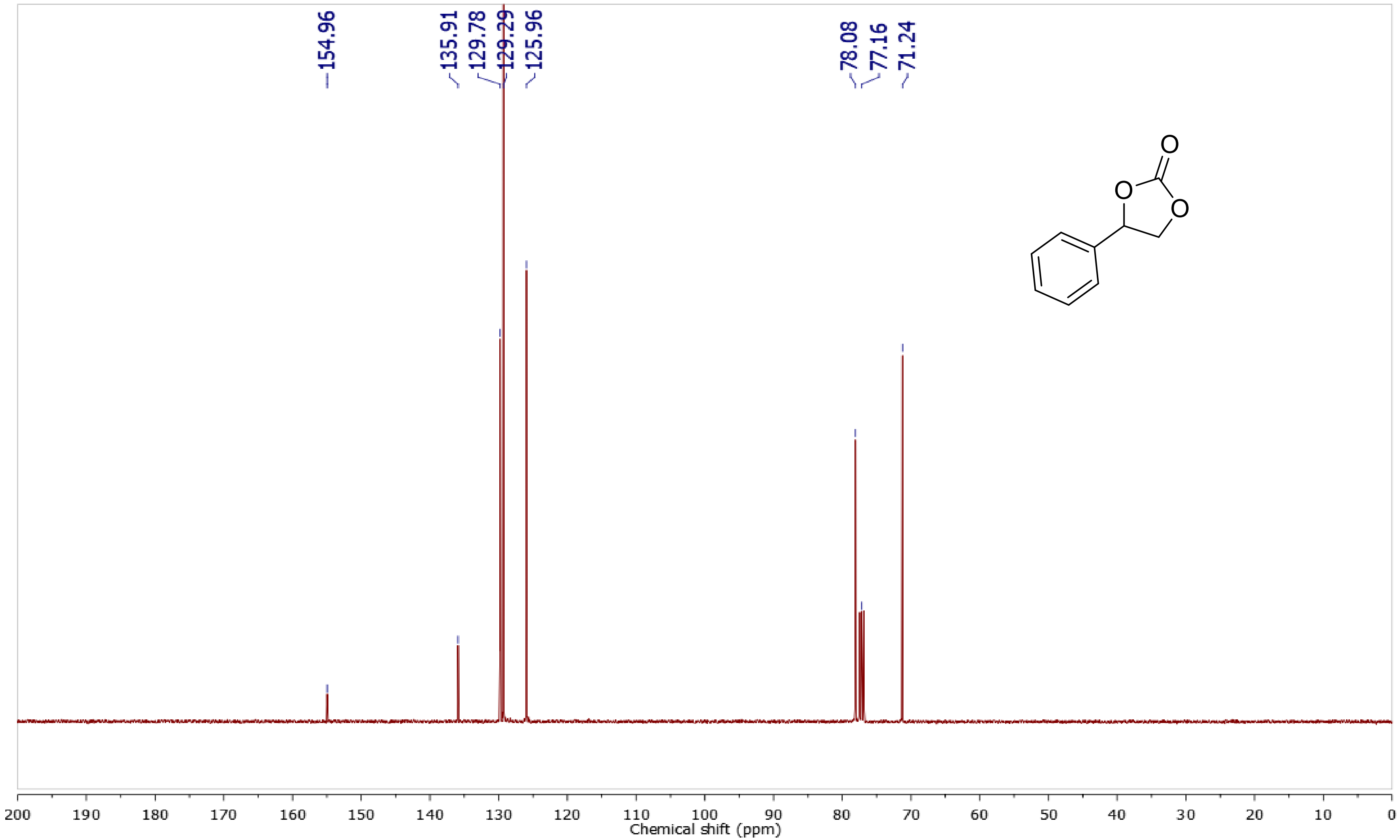

Figure S22. ^{13}C NMR of styrene carbonate in $CDCl_3$.

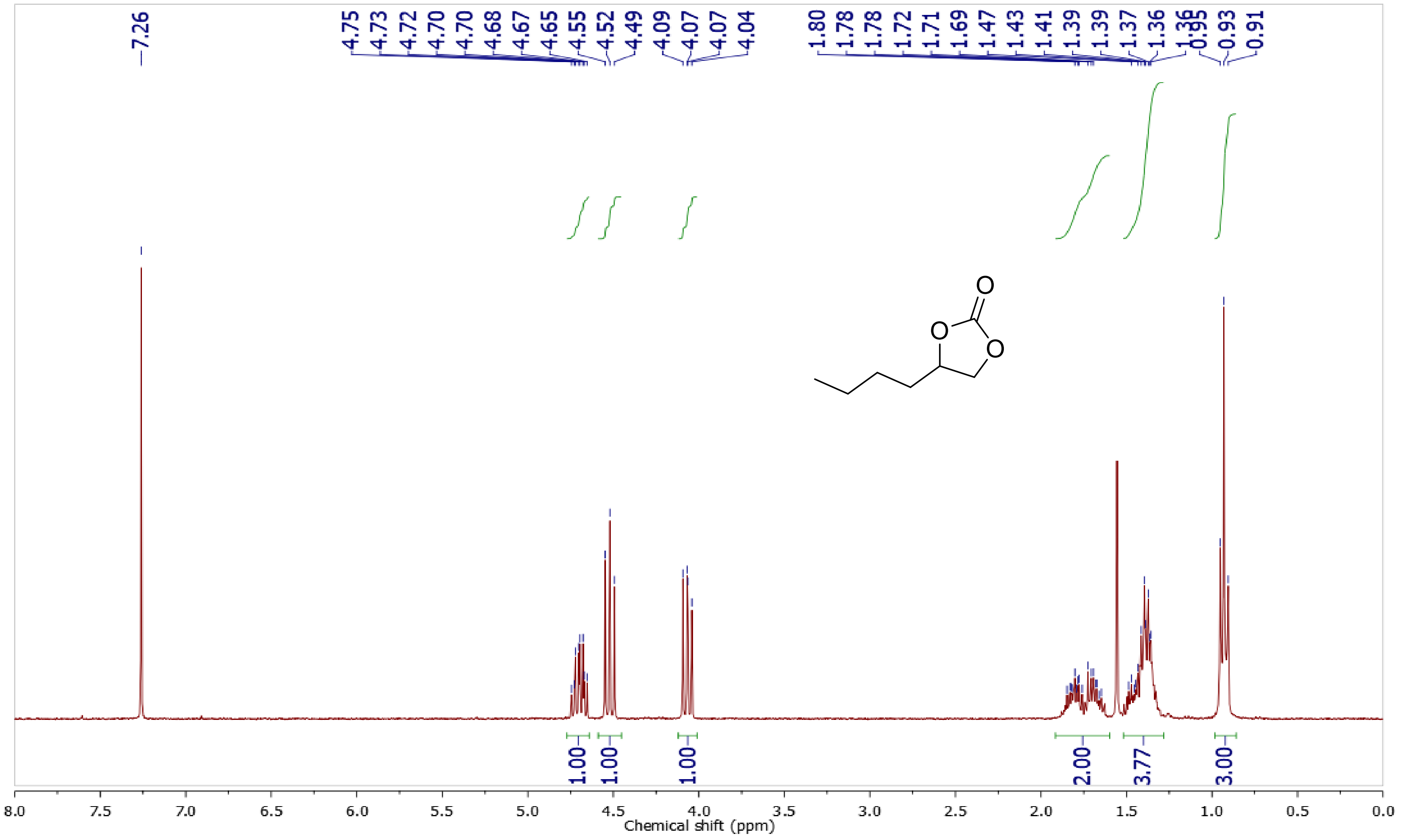

Figure S23. ^{1}H NMR of 1,2-hexylene carbonate in $CDCl_3$.

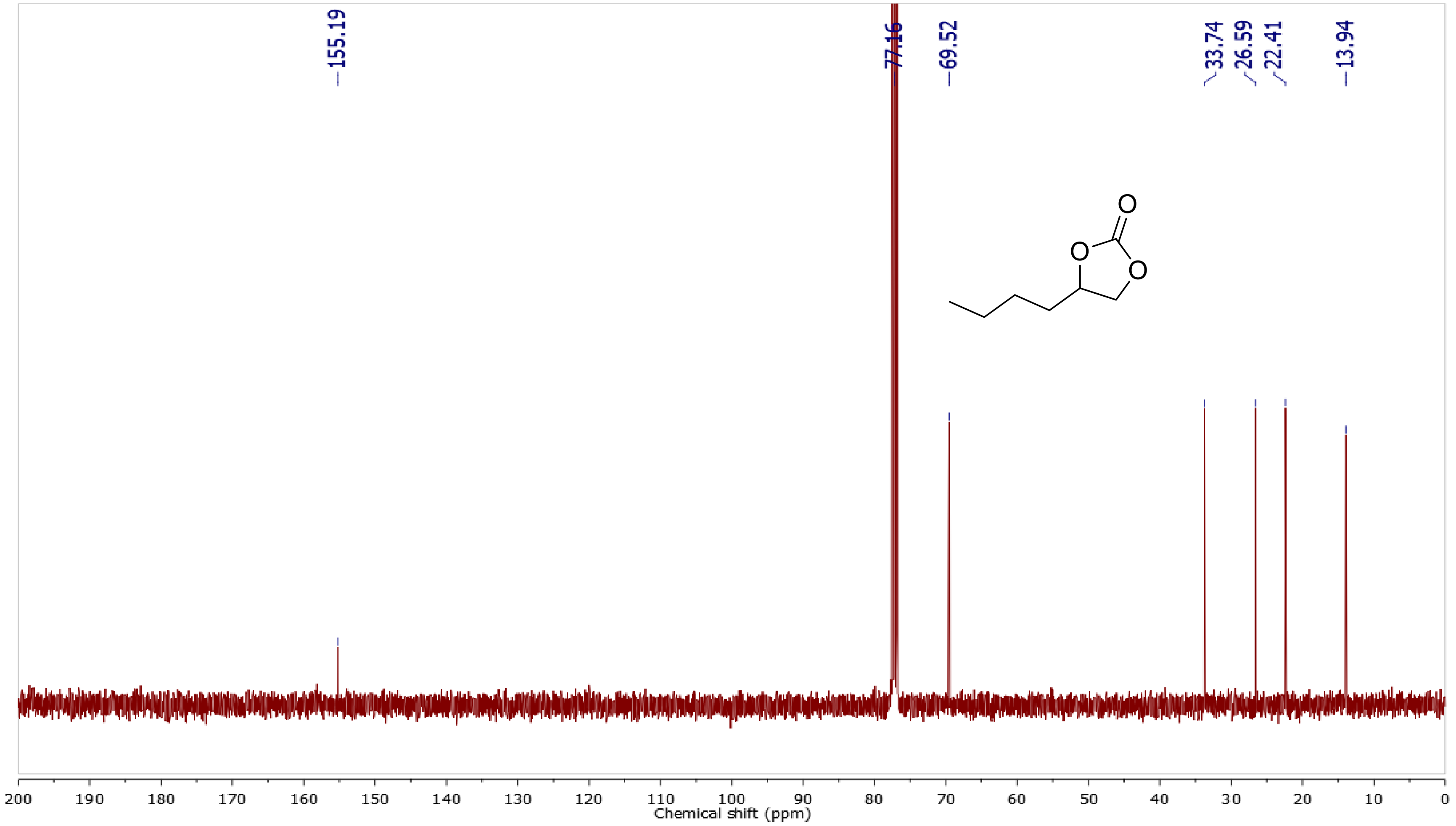

Figure S24. ^{13}C NMR of 1,2-hexylene carbonate in $CDCl_3$. A DEPT spectrum showed that the CHO peak was coincident with the $CHCl_3$ peak at 77.2 ppm (see Figure S23)

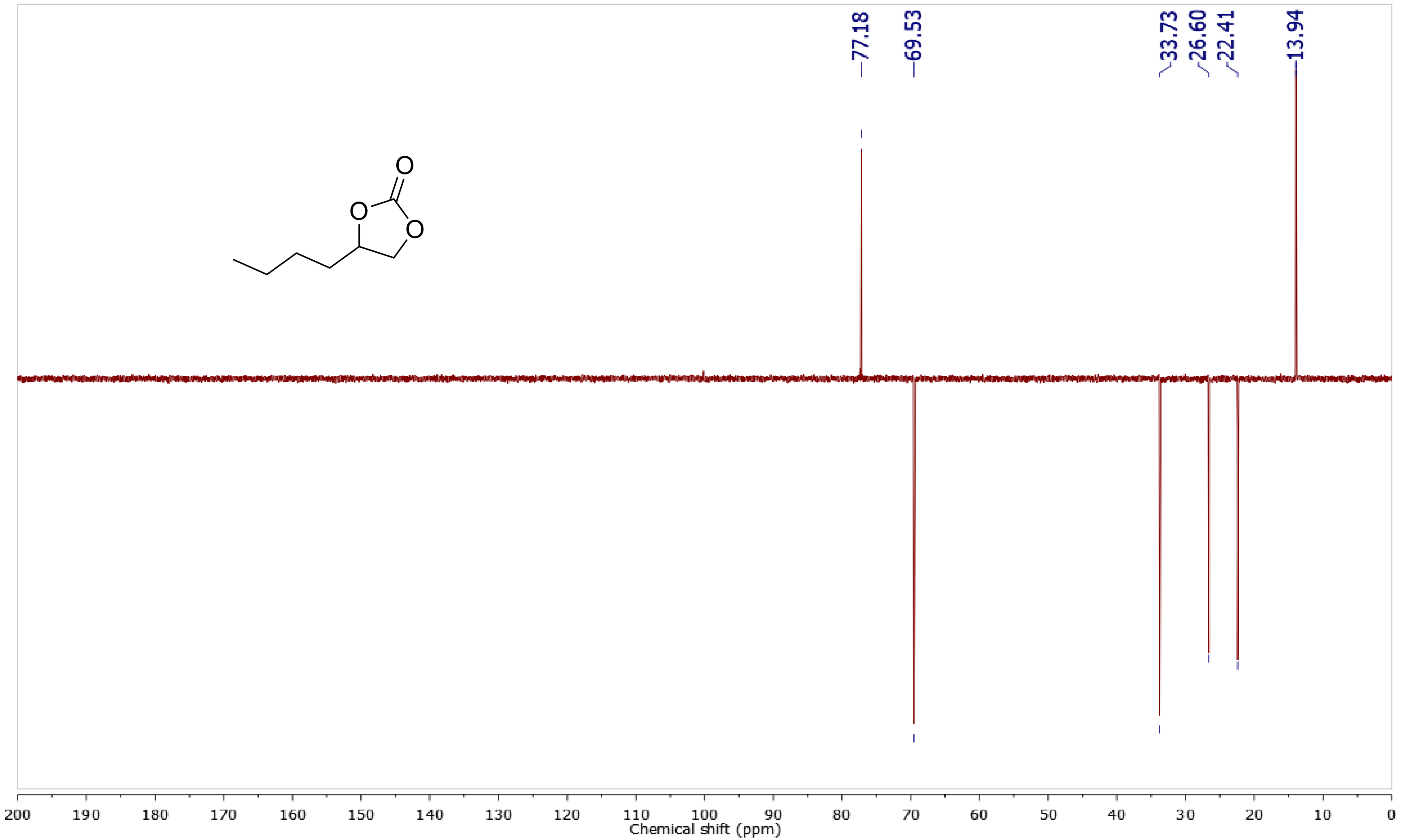

Figure S25. ^{13}C DEPT-135 NMR of 1,2-hexylene carbonate in $CDCl_3$.

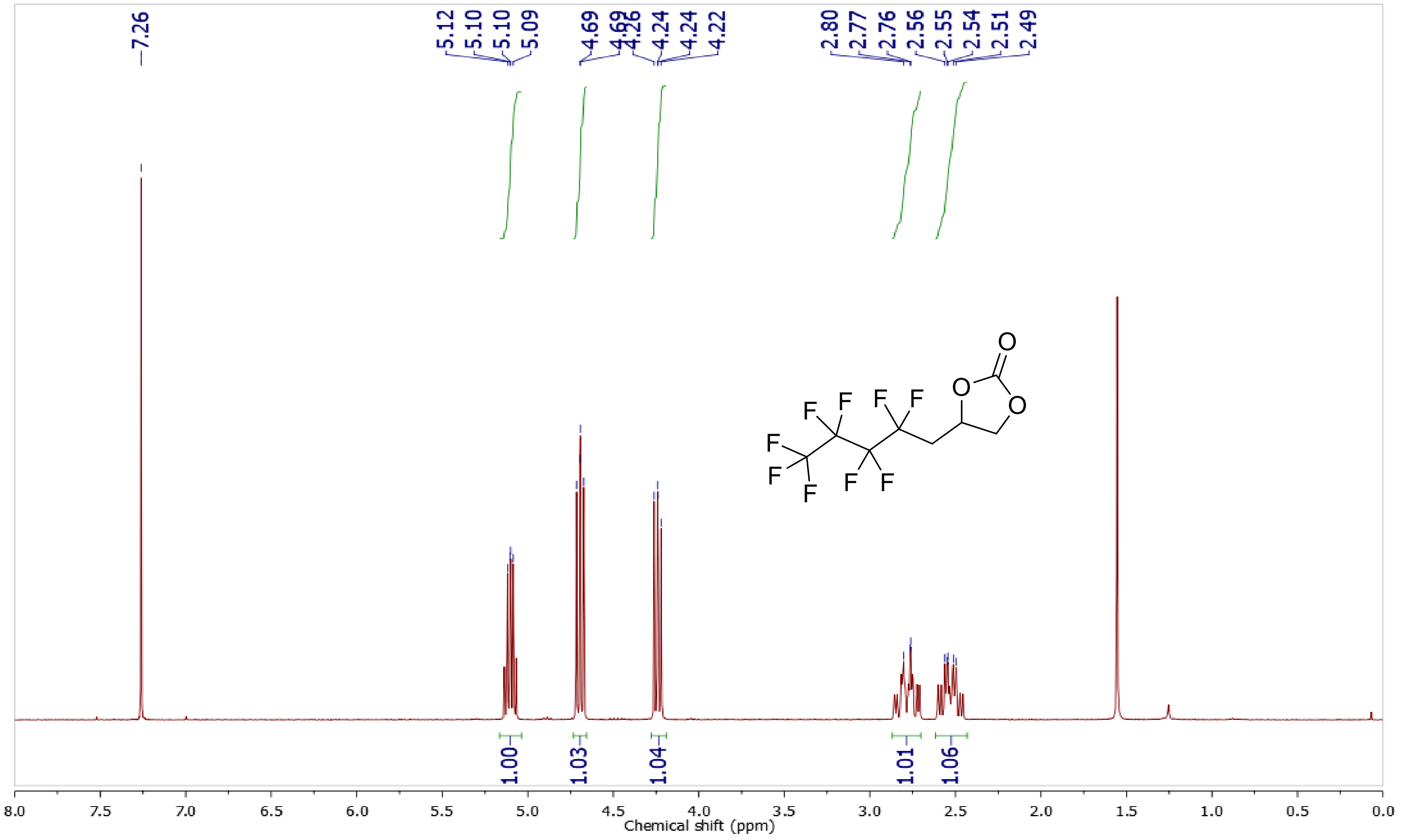

Figure S26. ^{1}H NMR of 2,2,3,3,4,4,5,5,5-nonafluoropentyl carbonate in $CDCl_3$.

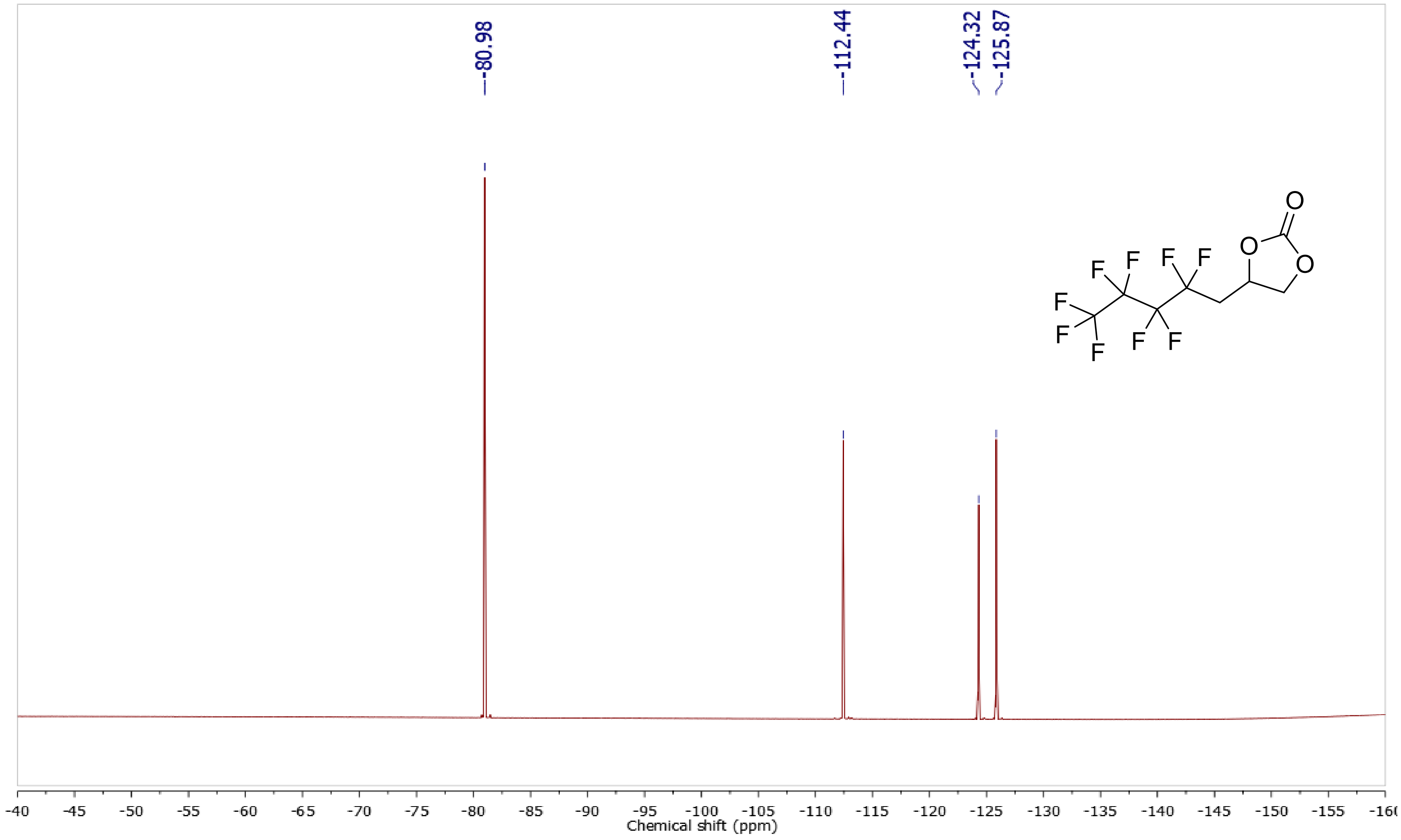

Figure S27. ^{19}F NMR of 2,2,3,3,4,4,5,5,5-nonafluoropentyl carbonate in $CDCl_3$.

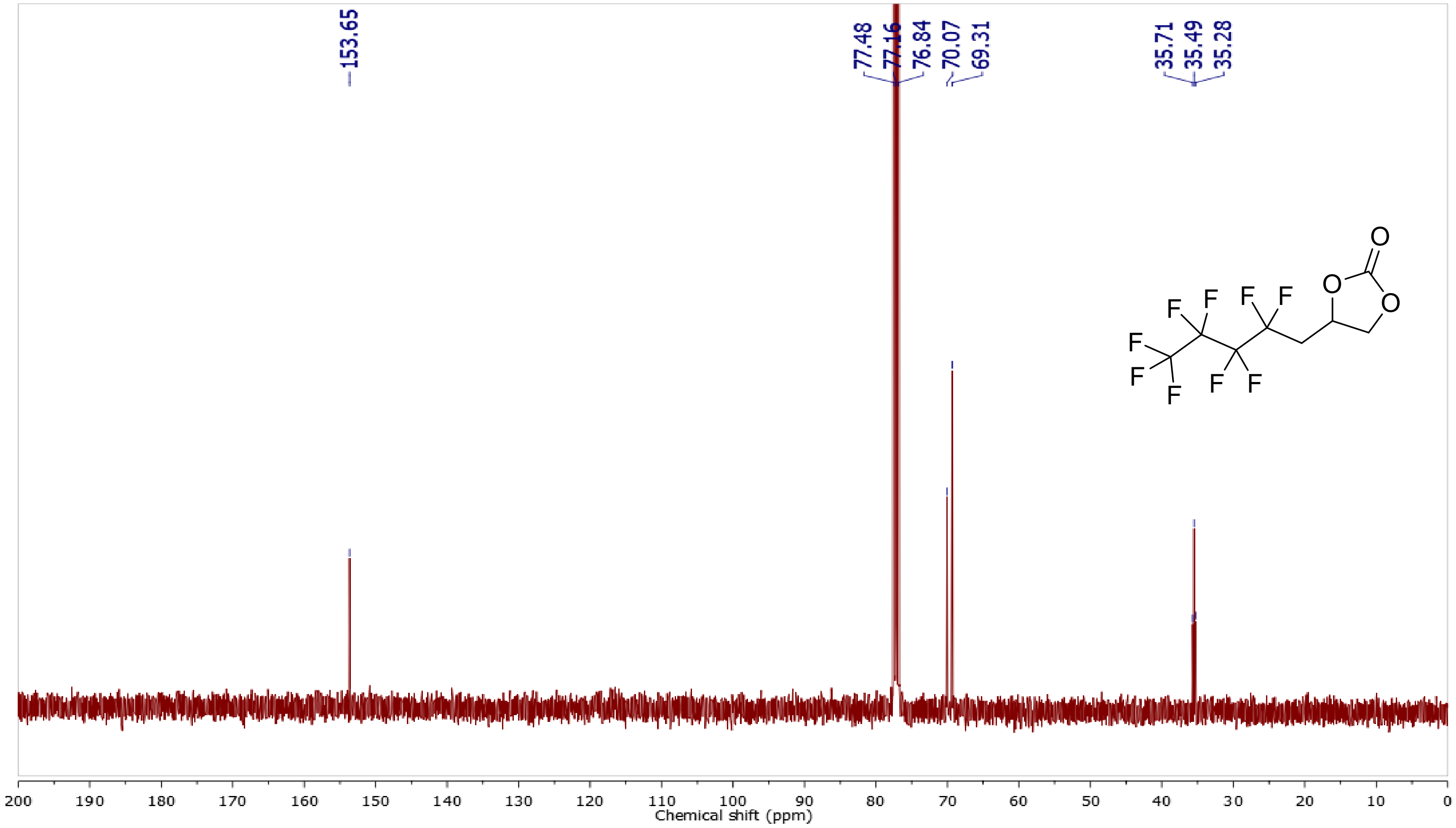

Figure S28. ^{13}C NMR of 2,2,3,3,4,4,5,5,5-nonafluoropentyl carbonate in $CDCl_3$. The CF_3 and CF_2 carbons cannot be clearly seen due to complicated splitting with F.[7]

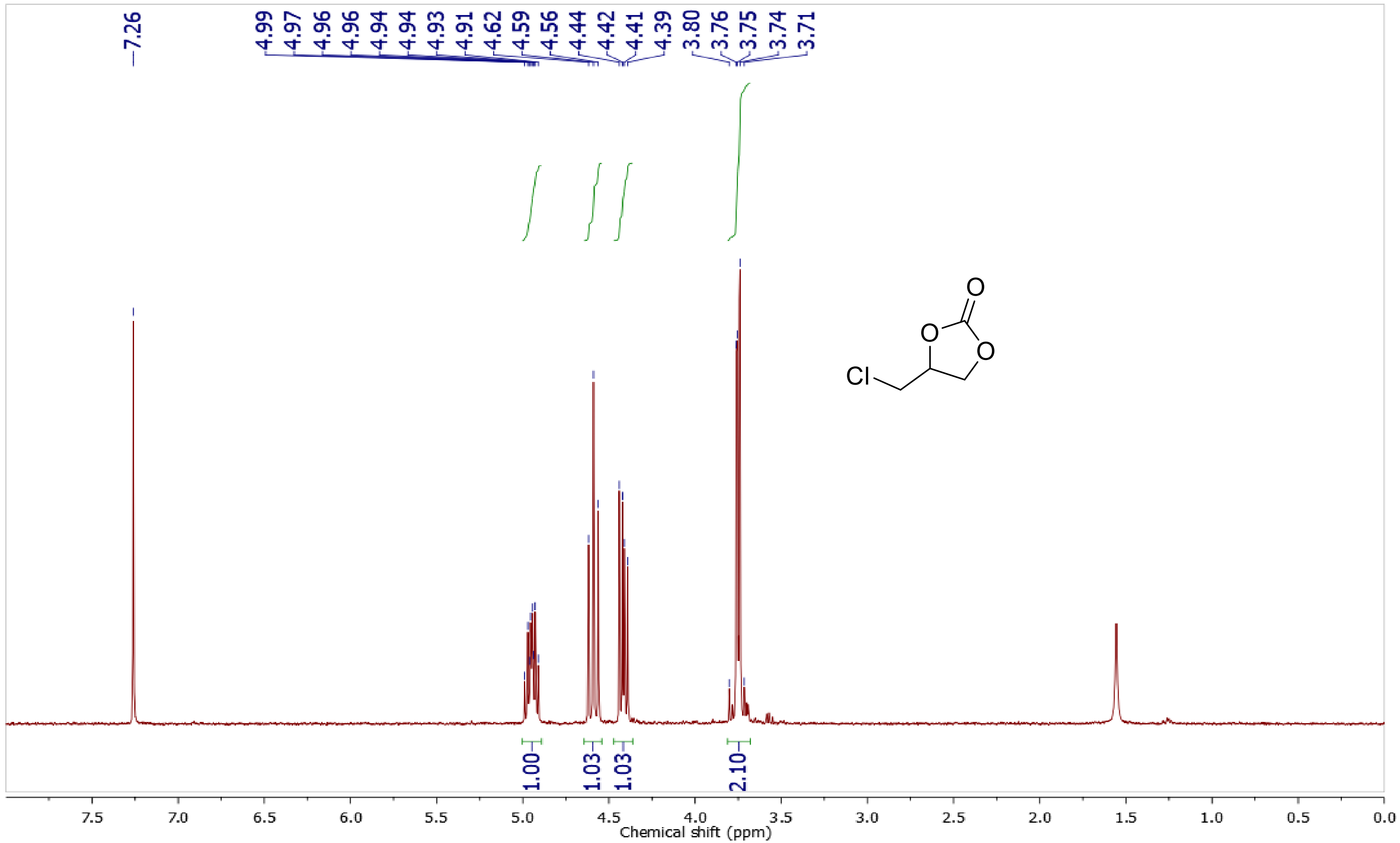

Figure S29. ^{1}H NMR of 3-chloropropylene carbonate in $CDCl_3$.

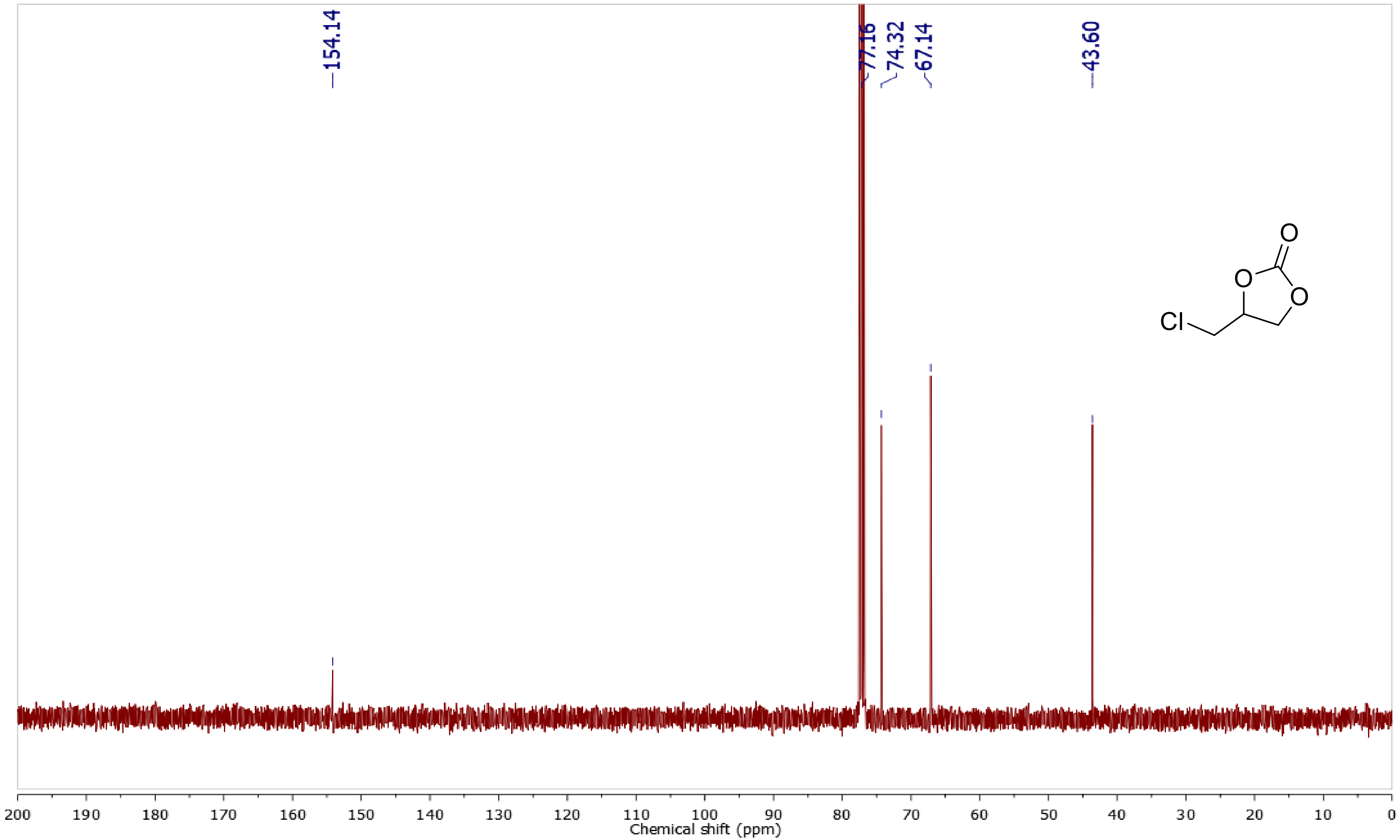

Figure S30. ^{13}C NMR of 1 3-chloropropylene carbonate in $CDCl_3$.

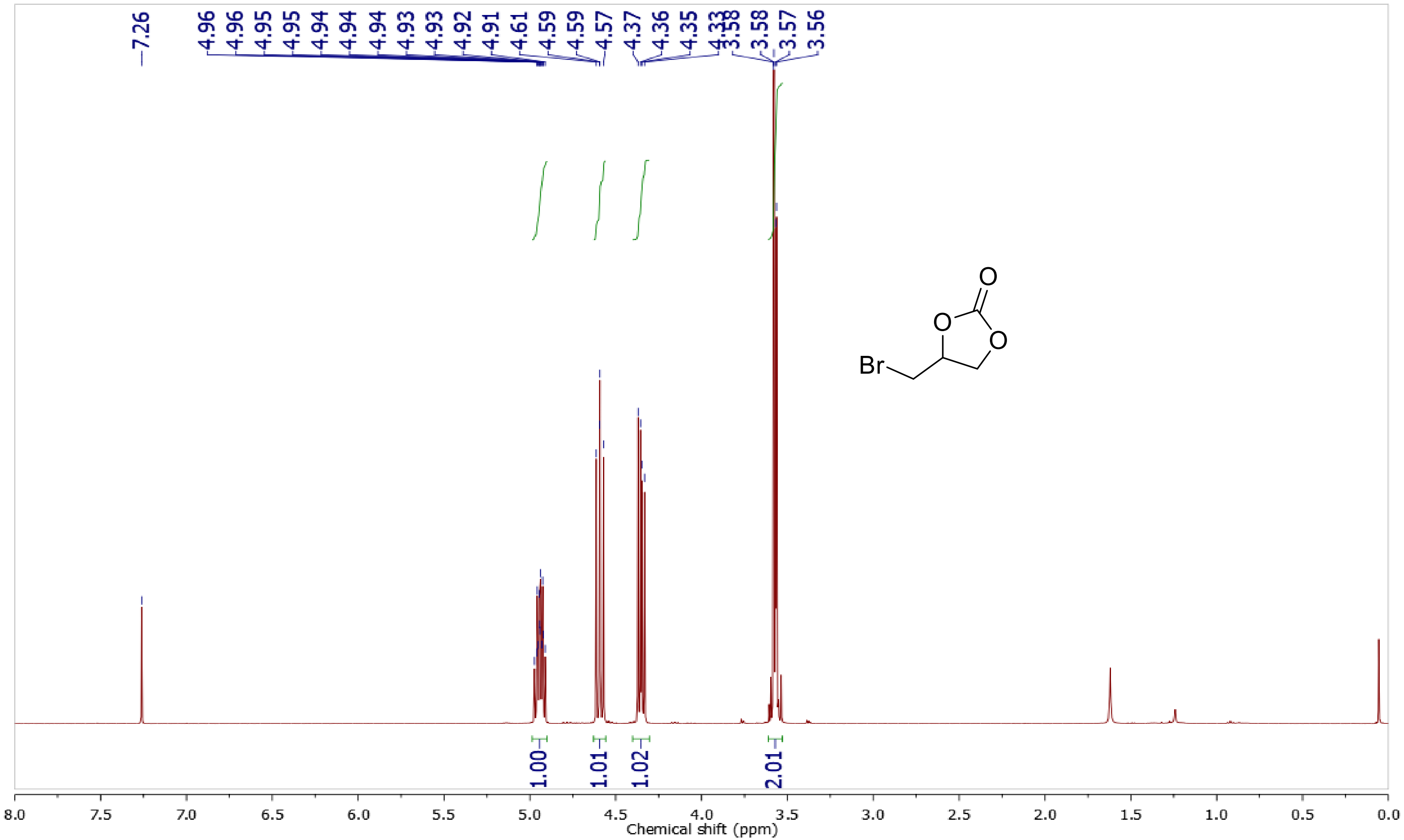

Figure S31. ^{1}H NMR of 3-bromopropylene carbonate in $CDCl_3$.

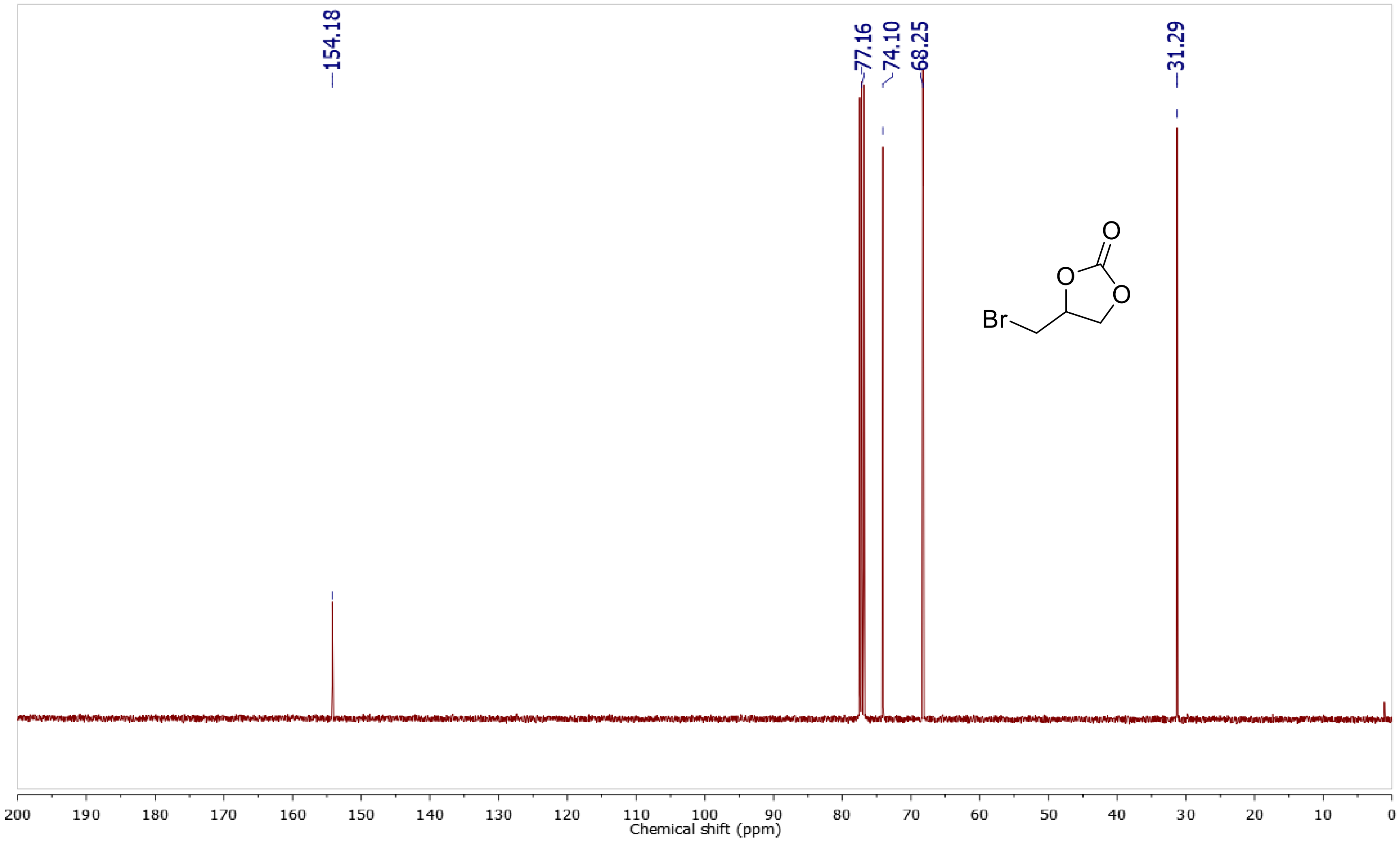

Figure S32. ^{13}C NMR of 3-bromopropylene carbonate in $CDCl_3$.

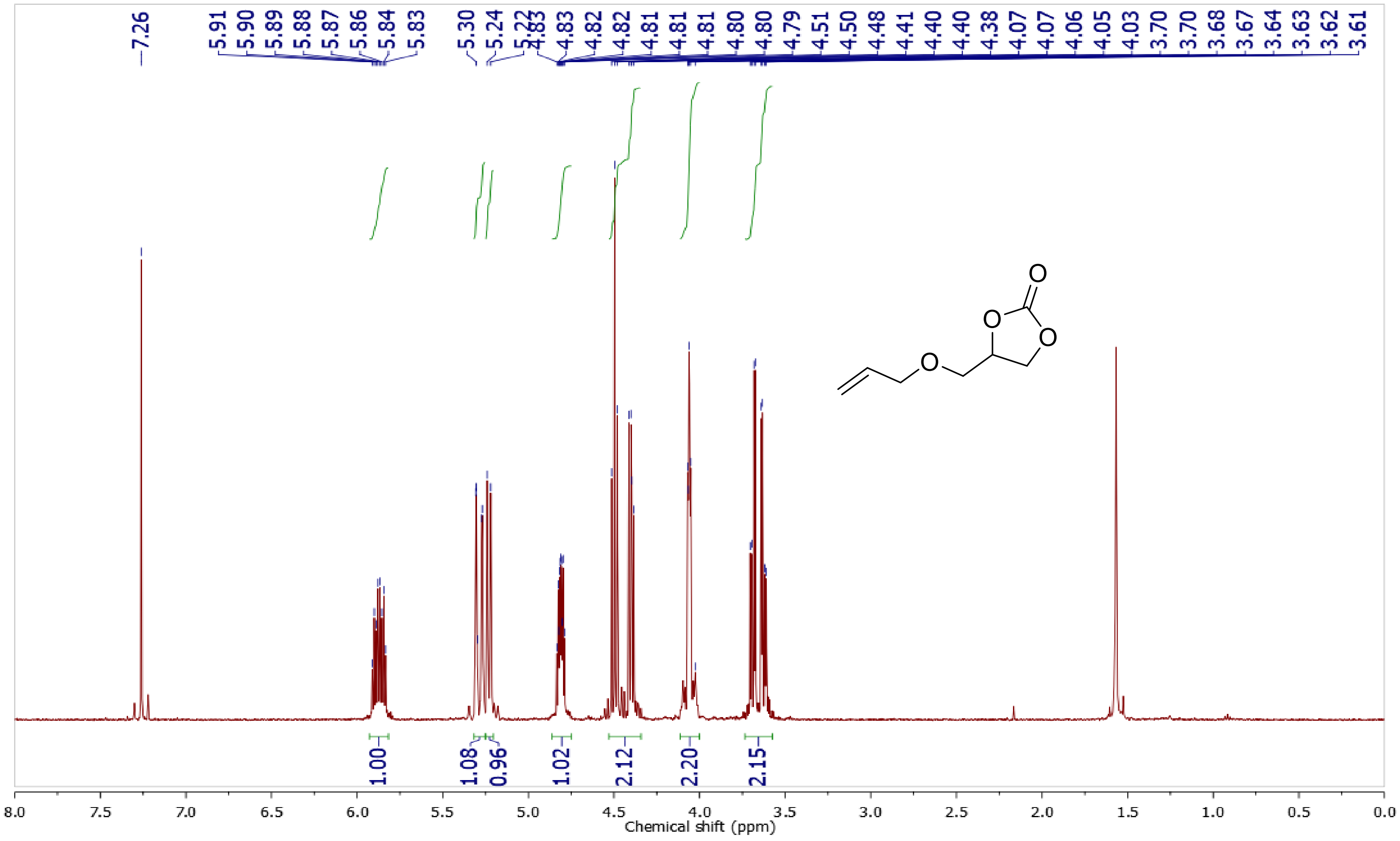

Figure S33. ^{1}H NMR of allyl glycidyl carbonate in $CDCl_3$.

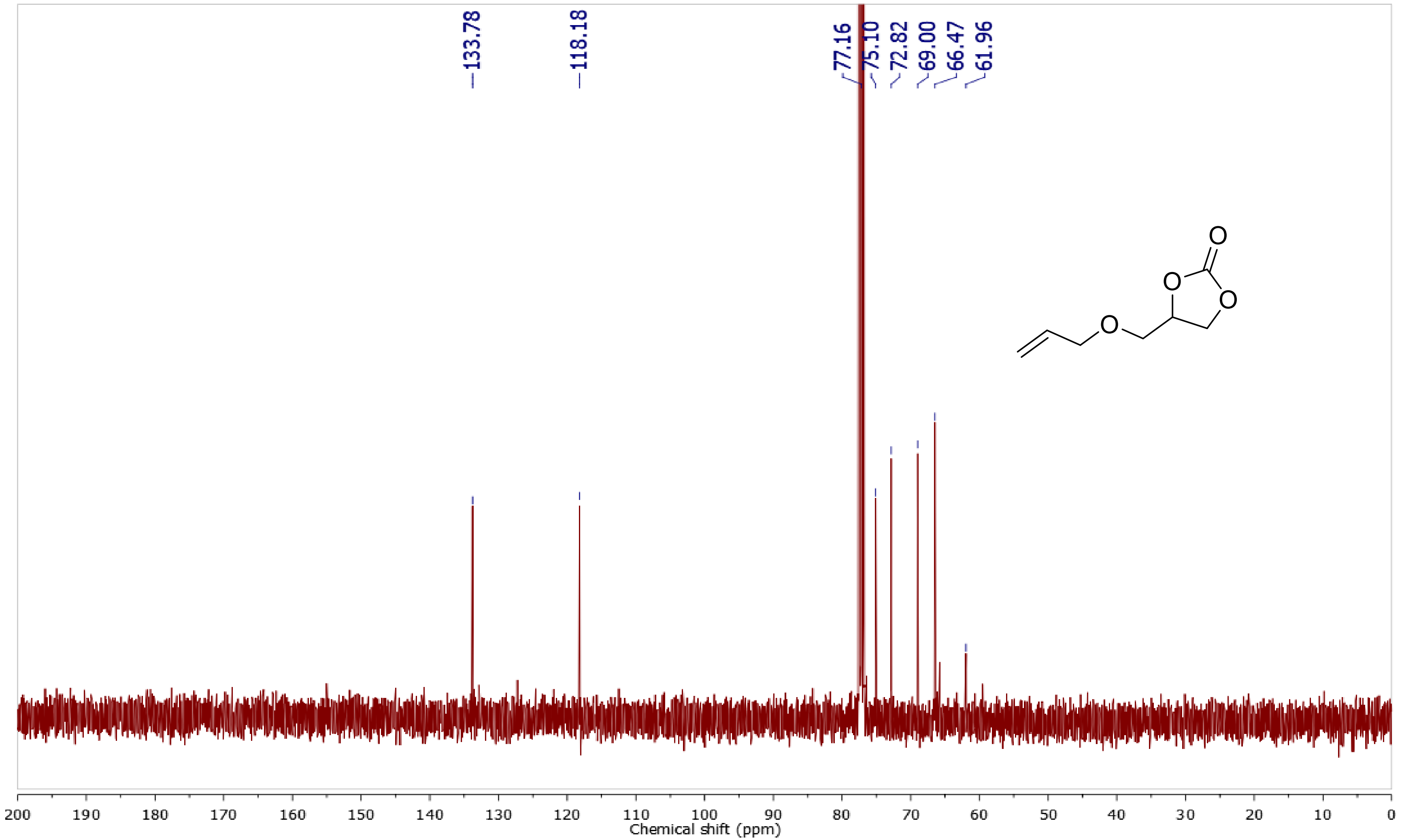

Figure S34. ^{13}C NMR of allyl glycidyl carbonate in $CDCl_3$.

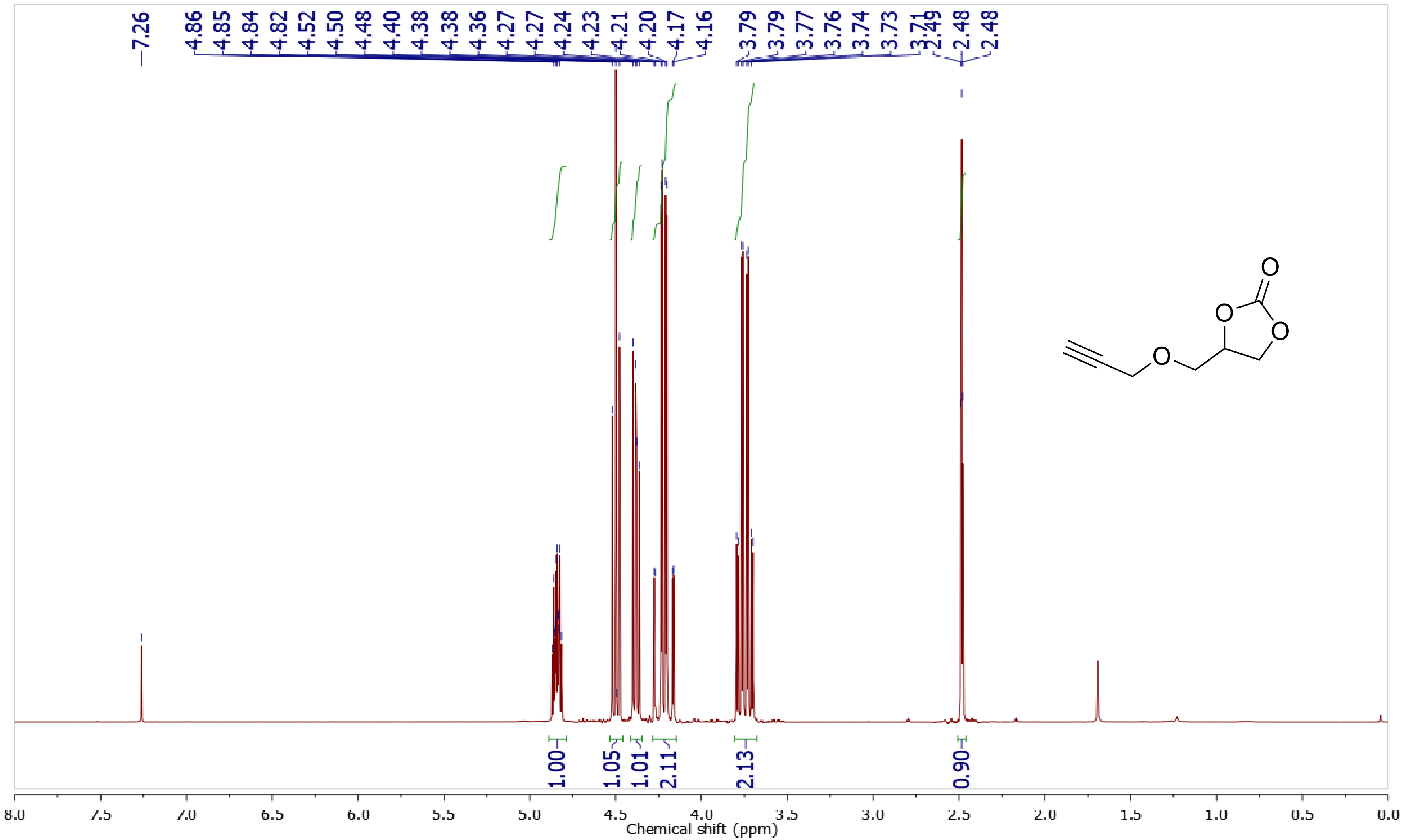

Figure S35. ^{1}H NMR of glycidyl propargyl carbonate in $CDCl_3$.

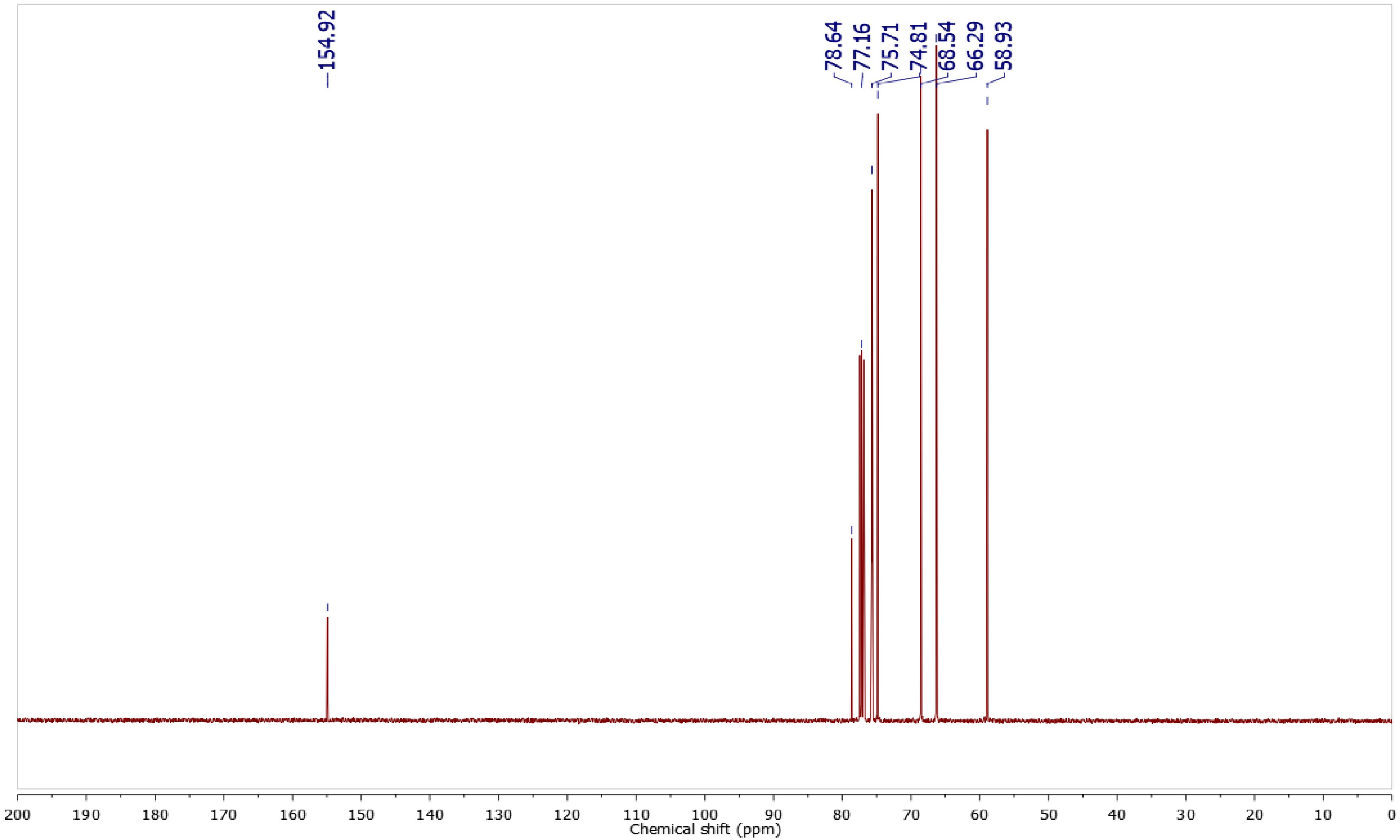

Figure S36. ^{13}C NMR of glycidyl propargyl carbonate in $CDCl_3$.

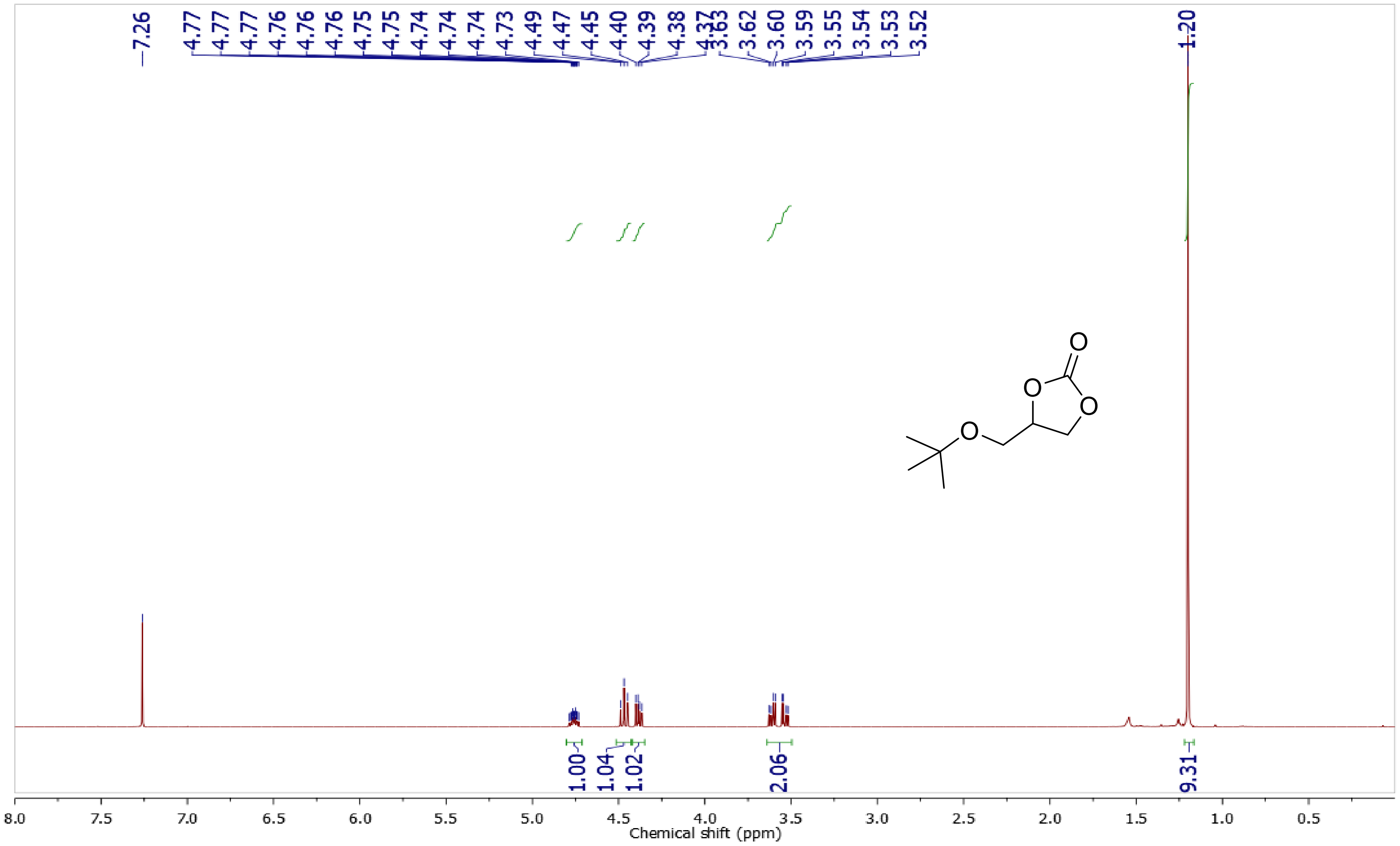

Figure S37. ^{1}H NMR of tert-butyl glycidyl carbonate in $CDCl_3$.

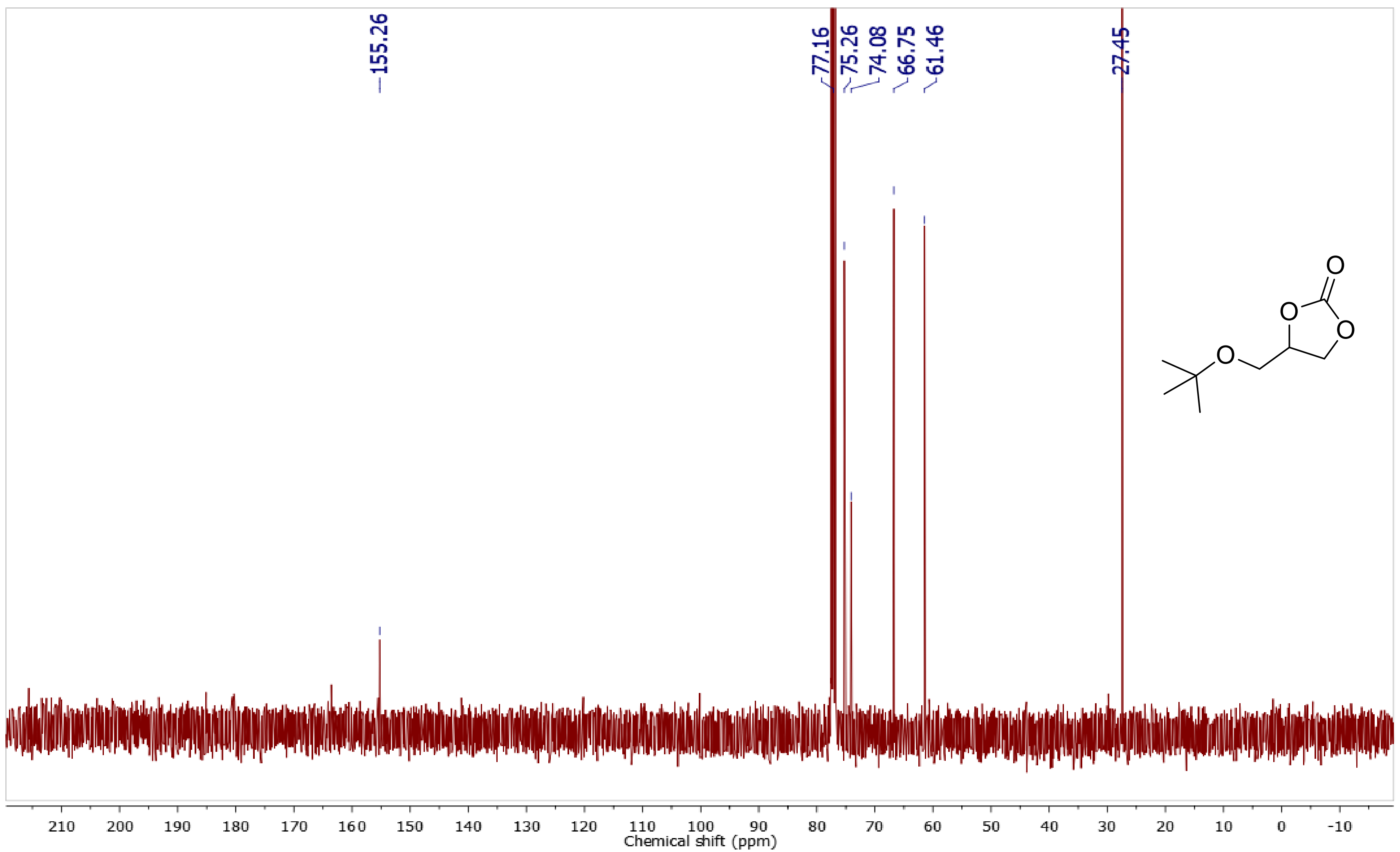

Figure S38. ^{13}C NMR of tert-butyl glycidyl carbonate in $CDCl_3$.

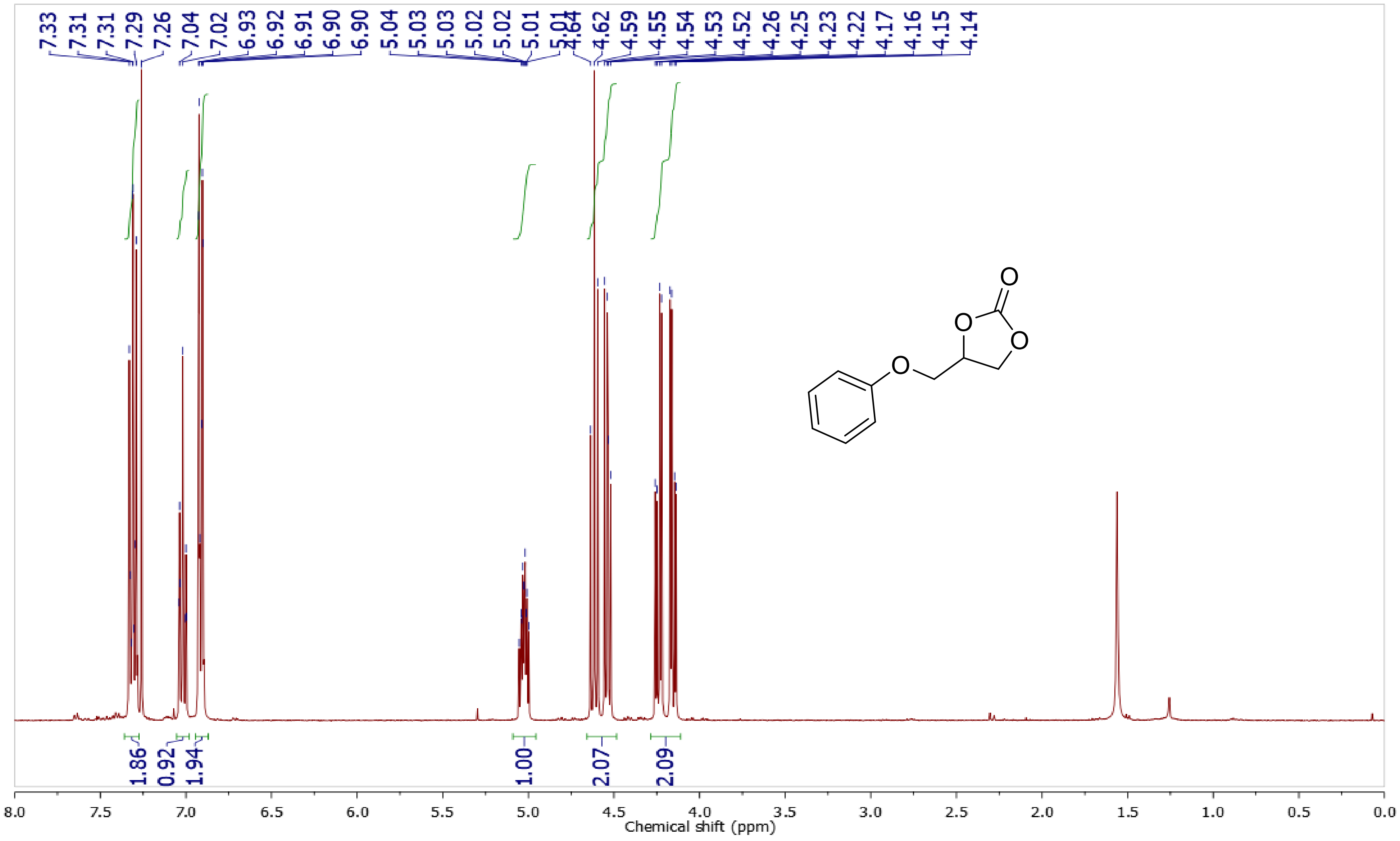

Figure S39. ^{1}H NMR of glycidyl phenyl carbonate in $CDCl_3$.

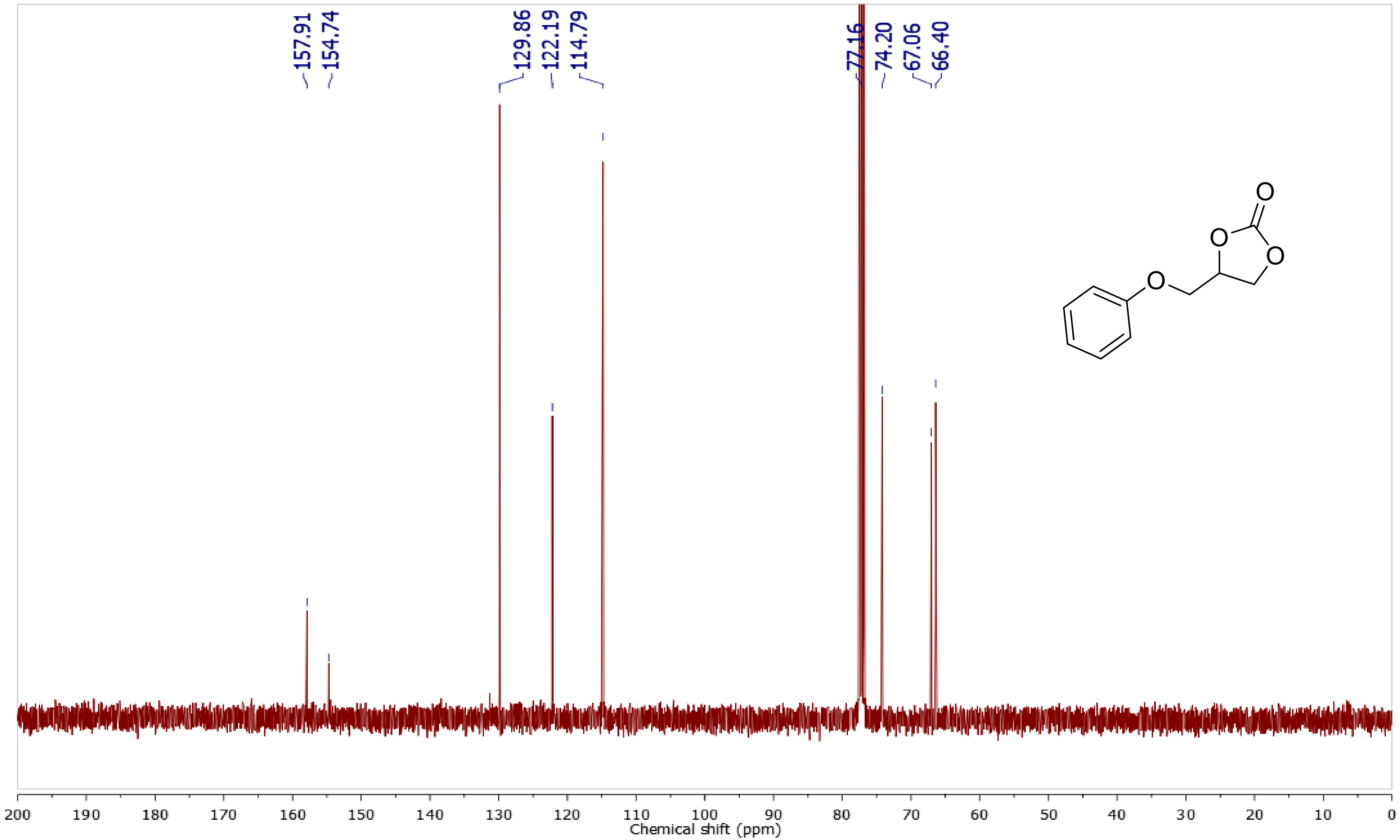

Figure S40. ^{13}C NMR of glycidyl phenyl carbonate in $CDCl_3$.

S6. Computational methods

Al(III)@salen ($C_{102}H_{123}Al_3N_6O_9$) was built and optimized using Gaussian 09[8] DFT at the level of theory B3LYP/6-31G. Packing of the cage (supercell, a = 44, b = 46, c = 23 angstroms) was built in Materials Studio and using G09 DFT optimized single cage geometry.

Table S4. DFT (G09: B3LYP/6-31G) optimized Al(III)@salen ($C_{102}H_{123}Al_3N_6O_9$) geometry (co-ordinates)

	x	y	z
O	4.403	-3.337	-11.077
O	1.452	6.149	1.209
O	-5.289	-2.354	1.436
C	0.285	-0.637	-3.972
C	0.427	0.730	-4.266
C	-0.670	1.608	-4.337
C	-1.954	1.042	-4.276
C	-2.147	-0.317	-3.962
C	-1.021	-1.152	-3.827
C	1.477	-1.417	-3.516
C	1.315	2.489	3.223
C	-1.970	-1.228	3.705
C	1.532	-2.806	-3.470
C	2.562	-3.476	-2.759
C	3.566	-2.732	-2.063
C	3.621	-1.316	-2.246

C	2.570	-0.721	-2.933
C	2.302	2.741	2.281
C	2.410	4.010	1.662
C	1.477	5.053	1.965
C	0.613	4.878	3.103
C	0.532	3.599	3.652
C	-2.065	-2.502	3.081
C	-3.184	-2.978	2.408
C	-4.302	-2.094	2.300
C	-4.310	-0.899	3.085
C	-3.139	-0.479	3.767
C	-5.925	0.608	-0.997
C	3.474	4.217	0.731
C	2.480	-4.908	-2.637
C	2.073	0.147	3.775
C	1.037	1.098	3.684
C	-0.297	0.657	3.787
C	-0.605	-0.699	4.010
C	0.461	-1.595	4.201
C	1.798	-1.217	3.980
C	2.812	-2.255	3.628
C	-0.445	3.065	-4.071
C	-3.479	-0.754	-3.450
C	2.382	-3.418	3.003

C	3.266	-4.210	2.238
C	4.631	-3.813	2.077
C	5.150	-2.808	2.967
C	4.220	-2.037	3.667
C	0.817	3.652	-4.119
C	1.133	4.760	-3.296
C	0.153	5.297	-2.406
C	-1.216	4.932	-2.612
C	-1.455	3.814	-3.405
C	-4.030	-2.054	-3.638
C	-5.142	-2.538	-2.945
C	-5.678	-1.709	-1.897
C	-5.276	-0.337	-1.852
C	-4.179	0.111	-2.620
O	5.362	-4.376	1.116
O	0.516	6.075	-1.381
O	-6.532	-2.166	-0.981
C	-5.450	-0.021	3.084
C	2.503	5.191	-3.242
C	2.725	-5.338	1.543
C	2.512	-7.072	-0.116
C	2.887	-7.074	-1.643
C	4.028	-8.028	-2.063
C	3.974	-9.391	-1.351

C	3.956	-9.195	0.175
C	2.685	-8.436	0.588
N	3.281	-5.967	0.534
N	3.261	-5.664	-1.915
C	-8.531	2.153	0.162
C	-7.291	1.474	0.785
C	-7.500	0.951	2.254
C	-8.924	0.468	2.616
C	-10.037	1.339	2.007
C	-9.856	1.444	0.482
N	-6.531	-0.167	2.367
N	-6.769	0.334	-0.029
C	4.843	5.250	-0.949
C	4.496	6.094	-2.227
C	4.914	7.581	-2.204
C	6.291	7.814	-1.559
C	6.323	7.209	-0.144
C	6.138	5.686	-0.226
N	3.639	5.254	-0.058
N	3.023	5.969	-2.332
C	6.674	-2.614	3.133
C	7.301	-3.982	3.528
C	7.005	-1.599	4.253
C	7.336	-2.111	1.822

C	-5.820	-3.878	-3.314
C	-5.168	-4.529	-4.557
C	-5.760	-4.905	-2.153
C	-7.309	-3.585	-3.658
C	-2.364	5.664	-1.889
C	-2.335	5.307	-0.381
C	-3.750	5.268	-2.455
C	-2.209	7.199	-2.071
C	4.746	-0.459	-1.625
C	4.705	1.006	-2.130
C	6.137	-1.035	-2.007
C	4.578	-0.434	-0.083
C	-3.159	-4.349	1.701
C	-3.052	-4.126	0.169
C	-1.946	-5.208	2.142
C	-4.440	-5.158	2.039
C	-0.142	6.077	3.722
C	-0.915	5.675	5.001
C	-1.161	6.688	2.727
C	0.902	7.159	4.121
H	0.759	-3.404	-3.946
H	2.549	0.357	-2.969
H	2.964	1.947	1.949
H	-0.156	3.425	4.470

H	-1.175	-3.110	3.059
H	-3.158	0.474	4.290
H	4.218	3.416	0.681
H	1.642	-5.383	-3.160
H	3.093	0.459	3.581
H	-1.095	1.350	3.543
H	0.243	-2.634	4.414
H	1.328	-3.674	2.976
H	4.580	-1.211	4.270
H	1.617	3.205	-4.703
H	-2.468	3.442	-3.450
H	-3.556	-2.691	-4.375
H	-3.828	1.127	-2.469
H	3.168	4.767	-4.003
H	1.732	-5.667	1.869
H	1.449	-6.793	-0.051
H	1.975	-7.332	-2.200
H	4.986	-7.541	-1.843
H	3.976	-8.155	-3.152
H	4.845	-9.986	-1.652
H	3.082	-9.962	-1.655
H	4.850	-8.638	0.485
H	3.983	-10.163	0.692
H	1.811	-9.054	0.333

H	2.650	-8.277	1.674
H	-7.210	1.766	2.933
H	-9.003	0.444	3.711
H	-9.039	-0.563	2.261
H	-10.035	2.349	2.445
H	-11.009	0.893	2.249
H	-9.863	0.437	0.043
H	4.978	4.211	-1.284
H	4.974	5.596	-3.084
H	4.157	8.144	-1.645
H	4.899	7.952	-3.237
H	6.489	8.892	-1.521
H	7.095	7.367	-2.166
H	5.523	7.656	0.461
H	7.274	7.433	0.355
H	6.987	5.260	-0.782
H	6.156	5.227	0.771
H	7.122	-4.729	2.751
H	6.878	-4.346	4.472
H	8.384	-3.870	3.660
H	6.651	-0.590	4.012
H	8.092	-1.544	4.377
H	6.573	-1.898	5.216
H	6.963	-1.117	1.555

H	7.135	-2.794	0.995
H	8.422	-2.038	1.964
H	-4.123	-4.807	-4.373
H	-5.205	-3.870	-5.432
H	-5.712	-5.447	-4.806
H	-6.320	-5.807	-2.433
H	-6.191	-4.490	-1.241
H	-4.726	-5.200	-1.950
H	-7.383	-2.888	-4.502
H	-7.830	-3.154	-2.800
H	-7.816	-4.516	-3.941
H	-2.493	4.230	-0.237
H	-3.131	5.845	0.149
H	-1.376	5.581	0.058
H	-3.811	5.432	-3.537
H	-4.520	5.884	-1.976
H	-3.999	4.219	-2.250
H	-3.023	7.718	-1.550
H	-2.260	7.470	-3.133
H	-1.258	7.548	-1.666
H	3.804	1.536	-1.798
H	4.759	1.060	-3.224
H	5.572	1.541	-1.725
H	6.275	-1.016	-3.095

H	6.246	-2.062	-1.659
H	6.928	-0.424	-1.554
H	3.601	-0.017	0.190
H	5.360	0.190	0.370
H	4.655	-1.439	0.334
H	-2.134	-3.578	-0.077
H	-3.907	-3.560	-0.200
H	-3.023	-5.094	-0.348
H	-1.916	-5.344	3.229
H	-0.992	-4.774	1.816
H	-2.028	-6.199	1.681
H	-4.414	-6.125	1.522
H	-5.337	-4.620	1.731
H	-4.500	-5.352	3.117
H	-1.706	4.946	4.790
H	-1.393	6.567	5.422
H	-0.253	5.258	5.769
H	-1.660	7.548	3.192
H	-1.928	5.953	2.461
H	-0.664	7.019	1.815
H	0.391	8.026	4.558
H	1.468	7.495	3.249
H	1.604	6.766	4.866
H	1.423	1.149	-4.307

H	-2.829	1.675	-4.360
H	-1.160	-2.161	-3.452
H	-6.485	2.222	0.828
H	-8.376	2.239	-0.921
H	-5.660	1.658	-1.163
H	-5.362	0.874	3.710
H	-8.580	3.179	0.556
H	-10.685	2.002	0.029
Al	2.111	6.492	-0.537
Al	-6.783	-1.506	0.788
Al	4.689	-5.022	-0.547
O	2.473	8.219	-0.451
C	1.926	9.286	0.338
C	2.878	10.480	0.329
H	3.846	10.199	0.760
H	2.466	11.314	0.912
H	3.047	10.823	-0.697
O	6.093	-5.925	-1.121
C	7.482	-5.868	-0.753
C	8.191	-7.142	-1.208
H	7.753	-8.020	-0.719
H	9.259	-7.105	-0.962
H	8.086	-7.267	-2.292
O	-8.353	-2.204	1.187

C	-9.079	-3.322	0.647
C	-10.579	-3.117	0.849
H	-11.146	-3.976	0.467
H	-10.917	-2.217	0.323
H	-10.806	-2.997	1.914
H	1.752	8.952	1.371
H	0.950	9.589	-0.074
H	7.587	-5.743	0.334
H	7.953	-4.994	-1.229
H	-8.761	-4.244	1.157
H	-8.856	-3.445	-0.422

References:

1. (a) P. Mucha, G. Mlostoń, M. Jasiński, A. Linden and H. Heimgartner, *Tetrahedron: Asymmetry*, 2008, **19**, 1600-1607; (b) M. P. Lalonde, M. A. McGowan, N. S. Rajapaksa and E. N. Jacobsen, *J. Am. Chem. Soc.*, 2013, **135**, 1891-1894; (c) H. Li, B. Xu, J. He, X. Liu, W. Gao and Y. Mu, *Chem. Commun.*, 2015, **51**, 16703-16706.
2. J. Gawronski, K. Gawronska, K. Kacprzak and M. Kwit, *Współczesna Synteza Organiczna*, PWN, Warszawa, 2004.
3. (a) X. Zheng, C. W. Jones and M. Weck, *Chem. Eur. J.*, 2006, **12**, 576-583; (b) Y. Jin, B. A. Voss, A. Jin, H. Long, R. D. Noble and W. Zhang, *J. Am. Chem. Soc.*, 2011, **133**, 6650-6658; (c) J. Chun, S. Kang, N. Kang, S. M. Lee, H. J. Kim and S. U. Son, *J. Mater. Chem. A*, 2013, **1**, 5517-5523; (d) Z. Dai, Q. Sun, F. Chen, S. Pan, L. Wang, X. Meng, J. Li and F.-S. Xiao, *ChemCatChem*, 2016, **8**, 812-817.
4. S. M. Elbert, F. Rominger and M. Mastalerz, *Chem. Eur. J.*, 2014, **20**, 16707-16720.
5. (a) S. Laokroekkiat, M. Hara, S. Nagano and Y. Nagao, *Langmuir*, 2016, **32**, 6648-6655; (b) J. Li, Y. Fan, Y. Ren, J. Liao, C. Qi and H. Jiang, *Inorg. Chem.*, 2018, **57**, 1203-1212.
6. D. Patel, G. R. Kurrey, S. S. Shinde, P. Kumar, G.-J. Kim and S. S. Thakur, *RSC Advances*, 2015, **5**, 82699-82703.
7. M.-Y. Wang, Y. Cao, X. Liu, N. Wang, L.-N. He and S.-H. Li, *Green Chem.*, 2017, **19**, 1240-1244.
8. Gaussian 09, Revision A.02, M. J. Frisch, G. W. Trucks, H. B. Schlegel, G. E. Scuseria, M. A. Robb, J. R. Cheeseman, G. Scalmani, V. Barone, B. Mennucci, G. A. Petersson, H. Nakatsuji, M. Caricato, X. Li, H. P. Hratchian, A. F. Izmaylov, J. Bloino, G. Zheng, J. L. Sonnenberg, M. Hada, M. Ehara, K. Toyota, R. Fukuda, J. Hasegawa, M. Ishida, T. Nakajima, Y. Honda, O. Kitao, H. Nakai, T. Vreven, J. A. Montgomery, Jr., J. E. Peralta, F. Ogliaro, M. Bearpark, J. J. Heyd, E. Brothers, K. N. Kudin, V. N. Staroverov, R. Kobayashi, J. Normand, K. Raghavachari, A. Rendell, J. C. Burant, S. S. Iyengar, J. Tomasi, M. Cossi, N. Rega, J. M. Millam, M. Klene, J. E. Knox, J. B. Cross, V. Bakken, C. Adamo, J. Jaramillo, R. Gomperts, R. E. Stratmann, O. Yazyev, A. J. Austin, R. Cammi, C. Pomelli, J. W. Ochterski, R. L. Martin, K. Morokuma, V. G. Zakrzewski, G. A. Voth, P. Salvador, J. J. Dannenberg, S. Dapprich, A. D. Daniels, O. Farkas, J. B. Foresman, J. V. Ortiz, J. Cioslowski, and D. J. Fox, Gaussian, Inc., Wallingford CT, 2009
